# Bioactive Components from *Ampelopsis japonica* with Antioxidant, Anti-α-Glucosidase, and Antiacetylcholinesterase Activities

**DOI:** 10.3390/antiox11071228

**Published:** 2022-06-23

**Authors:** Jia-Hua Liang, Hsiang-Ru Lin, Chang-Syun Yang, Chia-Ching Liaw, I-Chou Wang, Jih-Jung Chen

**Affiliations:** 1 Institute of Traditional Medicine, School of Medicine, National Yang Ming Chiao Tung University, Taipei 112304, Taiwan; liangjia.ps08@nycu.edu.tw; 2 Department of Chemistry, College of Science, National Kaohsiung Normal University, Kaohsiung 82444, Taiwan; t3136@nknu.edu.tw; 3 Department of Pharmacy, School of Pharmaceutical Sciences, National Yang Ming Chiao Tung University, Taipei 112304, Taiwan; tim0619@nycu.edu.tw; 4 Ministry of Health and Welfare, National Research Institute of Chinese Medicine, Taipei 112026, Taiwan; liawcc@nricm.edu.tw; 5 Department of Biochemical Science and Technology, National Chiayi University, Chiayi 600355, Taiwan; 6 Department of Physical Medicine and Rehabilitation, Kaohsiung Veterans General Hospital Tainan Branch, Tainan City 710011, Taiwan; 7 Department of Food Nutrition, Chung Hwa University of Medical Technology, Tainan City 717302, Taiwan; 8 Department of Medical Research, China Medical University Hospital, China Medical University, Taichung 404332, Taiwan

**Keywords:** *Ampelopsis japonica*, solvent extracts, bioactive compounds, antioxidants, anti-α-glucosidase, AChE inhibition, molecular docking

## Abstract

The dried root of *Ampelopsis japonica* (Thunb.) Makino (*A. japonica*.) is a traditional medicine used to treat fever, pain, and wound healing. It exhibits anti-inflammatory, antitumor, antityrosinase, and antimelanogenic activities. In this paper, we used different solvent extracts from the root of *A. japonica* to determine their antioxidant activity. Acetone extract showed relatively strong antioxidant properties by 2,2′-azino-bis(3-ethylbenzothiazoline-6-sulfonic acid) (ABTS), 2,2-diphenyl-1-(2,4,6-trinitrophenyl)hydrazyl (DPPH), superoxide radical scavenging activity, and ferric reducing antioxidant power (FRAP) assays. In addition, these extracts also showed significant α-glucosidase and acetylcholinesterase (AChE) inhibitory activities. Acetone extract significantly inhibited α-glucosidase with an IC_50_ value of 8.30 ± 0.78 μg/mL, and ethanol extract remarkably inhibited AChE with an IC_50_ value of 37.08 ± 7.67 μg/mL. Using HPLC analysis and comparison with the chemical composition of various solvent extracts, we isolated seven active compounds and assessed their antioxidant, anti-α-glucosidase, and anti-AChE activities. Catechin (**1**), gallic acid (**2**), kaempferol (**3**), quercetin (**4**), resveratrol (**6**), and epicatechin (**7**) were the main antioxidant components in the root of *A. japonica*. According to the results of DPPH, ABTS, and superoxide radical scavenging assays, these isolates showed stronger antioxidant capacity than butylated hydroxytoluene (BHT). Moreover, **1**, **3**, **4**, euscaphic acid (**5**), **6**, and **7** also expressed stronger anti-α-glucosidase activity than the positive control acarbose, and all the isolated compounds had a good inhibitory effect on AChE. Molecular docking models and hydrophilic interactive modes for AChE assays suggest that **1** and **5** exhibit unique anti-AChE potency. This study indicates that *A. japonica* and its active extracts and components may be a promising source of natural antioxidants, α-glucosidase, and AChE inhibitors.

## 1. Introduction

Oxidation is a natural process that forms free radicals called reactive oxygen species (ROS) and reactive nitrogen species (RNS) through a series of intermediate byproducts [1]. Free radicals are very active atoms or molecules. Because they have unpaired electrons, they can easily snatch electrons from other molecules and cause damage. These free radicals cause oxidative damage to biological macromolecules and damage to physiological macromolecules such as DNA, proteins, and lipids [2]. Cardiovascular disease, diabetes, cancer, neurodegenerative diseases, Alzheimer’s disease, and inflammatory diseases may occur in the body when antioxidants are lacking to eliminate excess reactive free radicals [3]. In order to reduce the oxidative damage of reactive free radicals, many synthetic antioxidants such as butylated hydroxyanisole (BHA) and butylated hydroxytoluene (BHT) have strong antioxidant activities and have been widely used in commercial foods in the form of additives. In recent years, the use of synthetic antioxidants has been limited due to their possible carcinogenic and toxic effects [4]. Therefore, natural antioxidants present in food and other biological materials are of interest due to their safety and potential nutritional value [5]. Antioxidants in Chinese herbal medicine (CHM) are popular for their low toxicity and desirable pharmacological activity [6,7]. Among the secondary metabolites of CHM, polyphenols have important inhibitory activities on enzymes such as acetylcholinesterase (AChE) and α-glucosidase [8].

Diabetes is a metabolic disorder caused by high blood sugar. One of the goals of reducing hyperglycemia is to reduce the activity of α-glucosidase, which is responsible for the hydrolysis of carbohydrates. α-Glucosidase inhibitors delay the intestinal absorption of glucose, thereby limiting the fluctuation of postprandial blood glucose [9]. There are several antidiabetic medications, such as acarbose, voglibose, and miglitol, which reduce high blood sugar levels by inhibiting the activity of α-glucosidase. However, continued use of synthetic drugs often results in adverse side effects such as liver toxicity, abdominal cramps, diarrhea, and vomiting [10,11]. On the other hand, acetylcholinesterase inhibitors are the most suitable modern and primary treatments against neurodegenerative diseases [12]. It is also a key enzyme in one of the treatment strategies for Alzheimer’s disease (AD). Acetylcholinesterase inhibitors block the action of AChE, thereby increasing the level of acetylcholine in the brain, which would otherwise be hydrolyzed to acetic acid and choline [13]. Currently, AChE inhibitors such as galantamine, donepezil, and rivastigmine have been used for the treatment of AD [14]. However, the efficacy of these drugs is hindered by their side effects, e.g., hepatotoxicity, gastrointestinal disturbances, and hypotension [15]. Therefore, natural antioxidants may be favorable candidates for the treatment of related diseases such as neurodegenerative diseases and diabetes.

The dried root of *Ampelopsis japonica* (Thunb.) Makino is a traditional remedy for fever, pain, and wound healing. Numerous pharmacological properties of this material have been reported, such as anti-inflammatory, antitumor, antityrosinase, and antimelanogenic activities [16]. In this study, we investigated the effects of various solvent extracts and major bioactive compounds from the root of *A. japonica* on antioxidant, anti-α-glucosidase, and AChE inhibitory activities.

## 2. Materials and Methods

### 2.1. Chemicals and General Procedures

Gallic acid, Folin–Ciocalteu reagent, hydrogen peroxide solution, α-glucosidase, acetylthiocholine iodide, 2,2′-azino-bis(3-ethylbenzothiazoline-6-sulfonic acid), acetylcholinesterase, 5,5′-dithio-bis(2-nitrobenzoic) acid, 2,4,6-tripyridyl-*s*-triazine, and Trolox were bought from Sigma-Aldrich. Nitroblue tetrazolium, 2,2-diphenyl-1-(2,4,6-trinitrophenyl)hydrazyl, loroglucinol, and phenazine methosulphate were supplied by Tokyo Chemical Industry Co., Ltd. (Tokyo, Japan). Aluminum chloride, ferric chloride, and *p*-nitrophenyl-α-D-glucopyranoside were bought from Alfa Aesar. Potassium dihydrogenphosphate, sodium carbonate, potassium peroxodisulfate, disodium hydrogenphosphate, and sodium carbonate were bought from the Showa Chemical Industry Co., Ltd. (Tokyo, Japan). Sodium acetate, butyl hydroxytoluene, potassium acetate, and nicotinamide adenine dinucleotide were supplied by Acros Organics. Galanthamine hydrobromide was bought from MedchemExpress.

### 2.2. Preparation of A. japonica Extracts

Preparation of *A. japonica* extracts was carried out as previously described [6]. Samples were collected, air-dried, and ground to powder. 200 mL of different solvents (*n*-hexane, dichloromethane, chloroform, acetone, ethyl acetate, ethanol, methanol, and water) were added to 20 g of powder and incubated with shaking by orbital shakers for 24 h at room temperature. The extracts were filtered and condensed under reduced pressure at 38 °C.

### 2.3. Preparation of Active Compounds

The root (100 g) of *A. japonica* was pulverized and extracted 3 times with MeOH (3 × 500 mL, 3 d each). The MeOH extract was concentrated under reduced pressure at 37 °C to give a residue (Fraction M, 5.64 g). Fraction M (5.46 g) was separated by column chromatography (215 g of silica gel, 70–230 mesh; *n*-hexane/ethyl acetate gradient) to obtain 11 fractions: M1–M11. Part (120.6 mg) of Fraction M2 was further by semi-preparative HPLC (reversed-phase C18 silica gel; 0.2% acetic acid in water (*v*/*v*)/acetonitrile acetate gradient) to obtain **5** (5.3 mg) and **6** (10.2 mg). Part (160.4 mg) of Fraction M3 was further by semi-preparative HPLC (reversed-phase C18 silica gel; 0.2% acetic acid in water (*v*/*v*)/methanol acetate gradient) to afford **7** (8.45 mg). Part (220 mg) of Fraction M4 was further purified by semi-preparative HPLC (normal-phase silica gel; *n*-hexane/acetone acetate gradient) to afford **3** (9.24 mg), and **4** (10.3 mg). Part (182 mg) of Fraction M5 was further by semi-preparative HPLC (reversed-phase C18 silica gel; 0.2% acetic acid in water (*v*/*v*)/methanol acetate gradient) to obtain **2** (26.3 mg). Part (86.4 mg) of Fraction M7 was further by semi-preparative HPLC (reversed-phase C18 silica gel; 0.2% acetic acid in water (*v*/*v*)/acetonitrile acetate gradient) to obtain **1** (15.4 mg). The structures of **1**-**7** were identified by NMR spectra (Figures S1–S7).

### 2.4. Reversed-Phase HPLC 

The reversed-phase HPLC assay to measure the seven components was performed as previously described with minor modifications [17,18]. Reversed-phase separations were executed using a LiChrospher^®^ 100 RP-18 Endcapped (5 μm; column of dimensions 4.6 × 250 mm). Gradient separation using 0.2% acetic acid in water (*v*/*v*) (solvent A) and methanol (solvent B) as mobile phase was as follows: 0-8 min, isocratic elution with 3% B; 8–10 min, gradient elution from 3 to 5% B; 10–25 min, isocratic elution with 5% B; 25–30 min, gradient elution from 5 to 20% B; 30–35 min, isocratic elution with 20% B; 35–150 min, gradient elution from 20 to 100% B; 150–155 min, back to initial conditions at 3% B; and 155–160 min, isocratic elution with 3% B. The flow rate was 1.0 mL/min, and the injection volume was 50 μL at room temperature. Peaks were detected at 280 nm. Different compounds were identified by retention time. 

### 2.5. Determination of Total Phenolic Content (TPC)

TPC of different solvent extracts was determined in accordance with the method previously reported [19].

### 2.6. Determination of Total Flavonoid Content (TFC)

TFC of various solvent extracts was measured by the reference method [20].

### 2.7. DPPH Radical Scavenging Activity

This assay was determined using the procedure previously reported [21].

### 2.8. ABTS Radical Scavenging Assay

This assay was assessed using the procedure previously described [22].

### 2.9. Superoxide Radical Scavenging Assay

This assay was determined by the method previously described [23].

### 2.10. Ferric Reducing Antioxidant Power (FRAP) Assay

The FRAP assay was measured using the procedure previously described [24].

### 2.11. α-Glucosidase Inhibitory Activity Assay

α-Glucosidase inhibitory activity was measured according to the method previously described [25].

### 2.12. Acetylcholinesterase (AChE) Inhibitory Assay

AChE inhibitory assay was determined using the reference method, with minor changes [26]. Briefly, 140 μL of 0.1 M sodium phosphate buffer (pH 8.0), 10 µL of DTNB, 20 µL of sample, and 15 µL of AChE solution were added in a 96-well microplate and incubated for 10 min. The reaction was then initiated with the addition of 10 µL of acetylthiocholine iodide, followed by an additional 10 min incubation. The absorbance was evaluated at 405 nm using a spectrophotometer.

### 2.13. Molecular Modeling Docking Study

All calculations were performed by Discovery Studio 2019 (San Diego, CA, USA) software. This study was calculated using the method previously described [17,18].

### 2.14. Statistical Analysis

The data are displayed as mean ± SEM. Statistical analysis was performed using Student’s *t*-test. A probability of 0.05 or less was considered statistically significant.

## 3. Results

### 3.1. Determination of TPC, TFC, and Yields in Each Solvent Extract

Various solvent extracts from *A. japonica* were assessed for TPC, TFC, and yields (Table 1). The yields of *A. japonica* different solvent extracts ranged from 0.28 ± 0.08% (*n*-hexane extract) to 16.46 ± 0.31 % (water extract). There were significant differences in TPC of various solvent extracts, among which the acetone extract of *A. japonica* had the highest TPC content (142.89 ± 4.07 mg/g), in succeeding order of methanol (95.98 ± 6.68 mg/g) > ethanol (95.69 ± 7.80 mg/g) > ethyl acetate (79.09 ± 8.45 mg/g) > water (47.38 ± 2.18 mg/g) > chloroform (20.92 ± 1.47 mg/g) > dichloromethane (20.19 ± 1.99 mg/g) > *n*-hexane (15.01 ± 0.44 mg/g). There were obvious differences in TFC content of different solvent extracts, among which the chloroform extract had the highest TFC content (94.22 ± 1.34 mg/g), followed by ethyl acetate (80.05 ± 7.82 mg/g), acetone (71.72 ± 2.66 mg/g), dichloromethane (62.60 ± 3.51 mg/g), *n*-hexane (56.36 ± 3.86 mg/g), ethanol (22.75 ± 1.24 mg/g), methanol (5.72 ± 1.13 mg/g), and water (4.45 ± 1.21 mg/g).

### 3.2. DPPH Radical Scavenging Activity

The acetone extract (SC_50_ = 54.88 ± 4.04 μg/mL) showed strong DPPH radical scavenging activity, followed by methanol (SC_50_ = 84.73 ± 7.82 μg/mL), ethanol (SC_50_ = 87.12 ± 6.45 μg/mL), ethyl acetate (SC_50_ = 92.14 ± 8.12 μg/mL), and water (SC_50_ = 98.54 ± 7.09 μg/mL), as shown in Table 2. In addition, the extracts of *n*-hexane, chloroform, and dichloromethane had no significant effect (SC_50_ > 200 μg/mL).

### 3.3. ABTS Free Radical Scavenging Effect

The acetone extract (SC_50_ = 33.88 ± 2.13 μg/mL) displayed the strongest ABTS radical scavenging activity, in succeeding order of methanol (SC_50_ = 53.77 ± 4.65 μg/mL), ethyl acetate (SC_50_ = 57.45 ± 4.74 μg/mL), ethanol (SC_50_ = 64.56 ± 4.80 μg/mL), and water (SC_50_ = 99.30 ± 7.02 μg/mL), as shown in Table 2. In addition, dichloromethane, chloroform, and *n*-hexane extracts had no significant effect (SC_50_ > 200 μg/mL).

### 3.4. Superoxide Radical Scavenging Effect

From the result in Table 2, all extracts of *A. japonica* were tested for their superoxide radical scavenging activity. Notably, the results exhibited that, except for methanol (SC_50_ = 290.83 ± 15.23 μg/mL), ethanol (SC_50_ = 307.20 ± 22.39 μg/mL), and water (SC_50_ = 313.84 ± 20.24 μg/mL), other extracts had no significant effect (SC_50_ > 400 μg/mL). 

### 3.5. Ferric Reducing Antioxidant Power (FRAP) Effect 

The acetone extract displayed the highest FRAP value (1001.00 ± 46.17 TE mM/g), followed by ethanol (736.95 ± 14.40 TE mM/g), methanol (712.56 ± 18.32 TE mM/g), ethyl acetate (587.11 ± 20.61 TE mM/g), and water (413.34 ± 21.08 TE mM/g) in Table 2. The solvent extracts with low polarities, such as dichloromethane, chloroform, and *n*-hexane, showed relatively weak FRAP values.

### 3.6. Anti-α-Glucosidase Effect

The anti-α-glucosidase activity of the acetone extract of *A. japonica* was the strongest (IC_50_ = 8.30 ± 0.78 μg/mL), followed by ethanol (IC_50_ = 11.06 ± 2.07 μg/mL), ethyl acetate (IC_50_ = 12.51 ± 2.42 μg/mL), methanol (IC_50_ = 19.27 ± 1.12 μg/mL), dichloromethane (IC_50_ = 28.00 ± 0.14 μg/mL), *n*-hexane (IC_50_ = 28.43 ± 3.78 μg/mL), and chloroform (IC_50_ = 34.16 ± 3.88 μg/mL), as shown in Table 3. Most various solvent extracts showed more potent anti-α-glucosidase activity than acarbose (IC_50_ = 335.50 ± 2.14 μg/mL). These results indicated that various solvent extracts of *A. japonica* had strong α-glucosidase inhibitory effects, except the water extract.

### 3.7. AChE Inhibition Activity

A previous study showed that chlorogenic acid has an AChE inhibitory effect [27]. Thus, chlorogenic acid was employed as a positive control. The ethanolic extract of *A. japonica* exhibited the highest inhibitory activity against AChE with an IC_50_ value of 37.08 ± 7.67 μg/mL, followed by acetone, methanol, *n*-hexane, water, dichloromethane, chloroform, and ethyl acetate, with IC_50_ values of 61.95 ± 5.54, 77.99 ± 5.08, 83.97 ± 8.90, 85.82 ± 8.74, 91.47 ± 26.03, 91.64 ± 8.77, and 103.30 ± 2.15 μg/mL, respectively (Table 3). All extracts showed good AChE inhibitory activity.

### 3.8. Quantitation of Active Components in Different Solvent Extracts

The HPLC methods using reversed-phase columns for the quantification of seven components isolated from *A. japonica* were verified regarding linearity, LOD, and LOQ. The linearity was validated by the data from six different concentrations (1.0, 5.0, 10.0, 25.0, 50.0, and 100.0 μg/mL) of the standard solutions. The linear regression parameters of calibration curves, correlation coefficient, LOD, and LOQ are shown in Table S1. Figure S8 shows the HPLC chromatogram of seven active ingredients.

**Figure 1:**
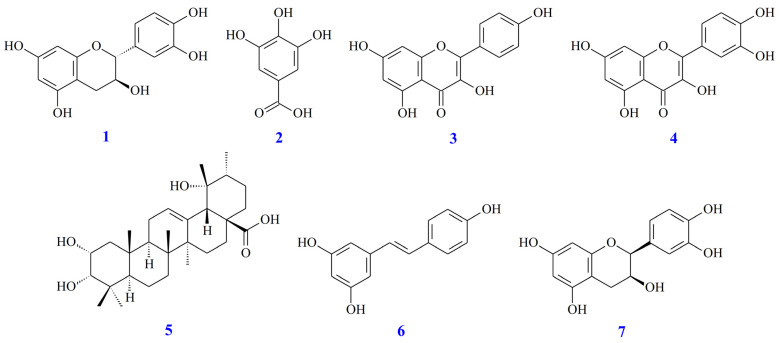
The chemical structures of catechin (**1**), gallic acid (**2**), kaempferol (**3**), quercetin (**4**), euscaphic acid (**5**), resveratrol (**6**), and epicatechin (**7**) from *Ampelopsis japonica*.

Figures S9–S16 shows the HPLC analyses of seven active ingredients [catechin (**1**), gallic acid (**2**), kaempferol (**3**), quercetin (**4**), euscaphic acid (**5**), resveratrol (**6**), and epicatechin (**7**)] (Figure 1) from different solvent extracts of *A. japonica* quantification (Table 4). Of all the extracts, the acetone extract had the highest content of the seven active ingredients. Of the seven active ingredients in all solvent extracts, compound **2** has the highest content.

### 3.9. Antioxidant Activities of Isolated Components

Seven isolated compounds were tested for antioxidant activity. The results are shown in Table 5, compound **2** (SC_50_ = 2.60 ± 0.67 μg/mL) showed the strongest DPPH radical scavenging activity, followed by **7** (SC_50_ = 2.78 ± 0.25 μg/mL), **4** (SC_50_ = 3.36 ± 0.58 μg/mL), **1** (SC_50_ = 10.08 ± 3.09 μg/mL), **3** (SC_50_ = 12.48 ± 3.01 μg/mL), and **6** (SC_50_ = 13.19 ± 4.78 μg/mL). There are six components showed stronger ABTS radical scavenging activities than BHT, in succeeding order of **2** (SC_50_ = 1.45 ± 0.14 μg/mL) > **1** (SC_50_ = 2.23 ± 0.22 μg/mL) > **6** (SC_50_ = 2.81 ± 0.12 μg/ mL) > **4** (SC_50_ = 3.15 ± 0.49 μg/mL) > **7** (SC_50_ = 3.78 ± 0.03 μg/mL) > **3** (SC_50_ = 5.24 ± 0.45 μg/mL). Compound **4** (SC_50_ = 31.89 ± 2.03 μg/mL) exhibited stronger superoxide radical scavenging activity than all isolated compounds. Furthermore, compound **2** (28,512.82 ± 43.27 mM TE/g), **4** (16,038.26 ± 86.89 mM TE/g), **7** (13,122.77 ± 182.42 mM TE/g), **1** (8729.33 ± 424.55 mM TE/g), **3** (7912.47 ± 220.08 mM TE/g), and **6** (7453.94 ± 60.09 mM TE/g) had higher antioxidant activity than BHT via FRAP assay.

### 3.10. Anti-α-Glucosidase Activities of Isolated Components

To further evaluate the inhibitory activity of α-glucosidase, the main components isolated from *A. japonica* were investigated. The results are shown in Table 6, compound **3** (IC_50_ = 5.81 ± 2.70 μg/mL), **4** (IC_50_ = 14.39 ± 5.93 μg/mL), **5** (IC_50_ = 20.38 ± 2.13 μg/mL), **6** (IC_50_ = 28.81 ± 5.65 μg/mL), **1** (IC_50_ = 81.78 ± 11.58 μg/mL), and **7** (IC_50_ = 88.73 ± 10.94 μg/mL) showed stronger α-glucosidase inhibitory activity than the positive control acarbose (IC_50_ = 334.53 ± 2.22 μg/mL).

### 3.11. AChE Inhibition Assay of Isolated Components

The inhibitory activity of seven main components from *A. japonica* against AChE is shown in Table 6. The results indicated that **5** (IC_50_ = 11.64 ± 2.69 μg/mL), **1** (IC_50_ = 26.35 ± 9.55 μg/mL), **2** (IC_50_ = 41.59 ± 7.57 μg/mL), **7** (IC_50_ = 53.38 ± 7.30 μg/mL), and **3** (IC_50_ = 55.04 ± 8.57 μg/mL) displayed stronger anti-AChE activity than chlorogenic acid (positive control) (IC_50_ = 64.42 ± 0.16 μg/mL).

### 3.12. Molecular Modeling Docking

The 3D crystal structure of the acetylcholinesterase complexed with acetylcholine (PDB: 2ACE) from *Torpedo californica* exhibits that the substrate binding site of acetylcholinesterase is formed by 14α-helices, 14β-sheets, and numerous loops in a gorge shape [28]. Once acetylcholine enters the substrate binding pocket by leaning its acetyl group toward the catalytic site (esteric site) and resides its trimethylamine group to the anionic site, it is surrounded by hydrophilic and hydrophobic residues including Trp 84, Gly 119, Glu 199, Ser 200, Phe 288, Phe 299, Glu 327, Phe 331, and His 440. Among these residues, Ser 200, Glu 327, and His 440 are viewed as the key residues at the esteric site to perform the hydrolytic reaction for acetylcholine when Ser 200 acts as the nucleophile. Furthermore, Trp 84 is considered as the essential residue at the anionic site. For the interactive binding mode of acetylcholine in the substrate binding pocket, the carbonyl group of acetylcholine is reduced to the hydroxyl group, which further interacts with the backbone of Gly 119 by acting as the H-bond acceptor. Apart from this H-bond interaction, there is no significant hydrophilic interaction between acetylcholine and the substrate binding pocket, especially the trimethylamine group of acetylcholine, which has been hypothesized to possibly make essential ionic interaction with the residues at the anionic site.

**Figure 2:**
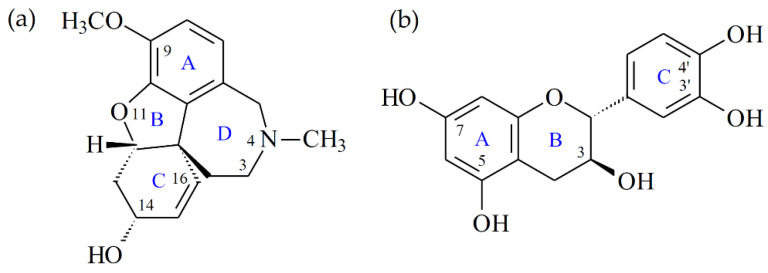
The chemical structures of galanthamine (**a**) and catechin (**1**) (**b**).

For the binding mode of galanthamine (Figure 2a) in the substrate binding pocket of acetylcholinesterase, the crystal structure of the acetylcholinesterase complexed with (−)-galanthamine (PDB: 1W6R) from *Torpedo californica* has been disclosed [29]. Galanthamine binds to the substrate binding pocket by leaning its A, B, and C rings at a similar position as the acetyl group of acetylcholine, and locates its D ring at the anionic site. Since the A, B, and C rings of galanthamine are nearby Ser 200 and His 440, their three hydrophilic groups can form hydrophilic interactions with the esteric site, including (1) the 9-methoxy group on the A ring interacts with Ser 200 by performing as the H-bond acceptor, (2) the oxygen atom on the B ring also serves as the H-bond acceptor to interact with Ser 200, and (3) the 14-hydroxyl group on the C ring contacts with Glu 199 by serving as the H-bond donor. Additionally, the D ring of galanthamine, containing a tertiary amino group, which does not exhibit obvious ionic interaction, employs the methyl substituent on the amino group to make the nonclassical H-bond interaction with Asp 72. More importantly, galanthamine makes several essential π–π interactions in the substrate binding pocket, including (1) the A ring of galanthamine interacts with Phe 331, (2) the C ring of galanthamine contacts with Trp 84, and (3) the D ring of galanthamine interacts with Trp 84 and Phe 330.

To further study the interaction between catechin (**1**) (Figure 2b) (or gallic acid (**2**)) and acetylcholinesterase, and try to interpret how **1** (or **2**) might exert its antagonistic effect, The crystal structure of the acetylcholinesterase from *Electrophorus electricus* (PDB: 1C2B) was also used in this study [30]. The crystal structures of acetylcholinesterase from *Electrophorus electricus* and *Torpedo californica* share high sequence homology and have very similar conformation. In the substrate binding site for both structures, the key residues, including Ser 203 (Ser 200 in *Torpedo californica*), His 447 (His 440 in *Torpedo californica*), Glu 334 (Glu 327 in *Torpedo californica*), Trp 86 (Trp 84 in *Torpedo californica*), Tyr 133 (Tyr 130 in *Torpedo californica*), Phe 338 (Phe 331 in *Torpedo californica*), and Tyr 449 (Tyr 442 in *Torpedo californica*), are almost identical. For the binding model of **2**, **2** resided in the substrate binding pocket by leaning its three hydroxyl substituents toward the esteric site and locating its benzoic acid moiety at the anionic site. Compound **2** made two H-bond interactions comprising (1) the 3-hydroxyl group docked with the backbone of Gly 120 by serving as the H-bond donor, and (2) the carboxylate group made H-bond contact with Tyr 124 and Ser 125 by performing as the H-bond acceptor. Additionally, **2** made the essential π–π interaction with Trp 86 at the anionic site.

**Figure 3:**
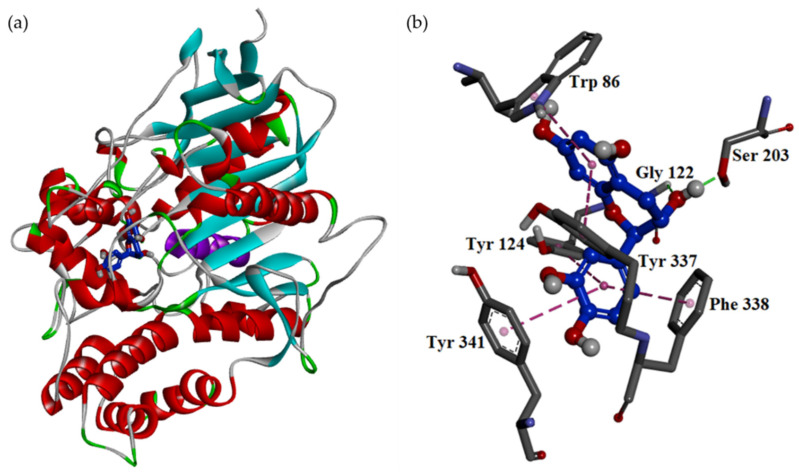
Interaction of catechin (**1**) with the active sites of acetylcholinesterase from *Electrophorus electricus*. The binding mode (**a**) and the hydrophilic interactive model (**b**) of catechin (**1**) in the substrate binding pocket of the crystal structure (PDB: 1C2B).

The docking model of catechin (**1**) exhibited that **1** resided at a similar position as galanthamine, as shown in Figure 3. In the substrate binding site, the B ring of **1** as the B ring of galanthamine leaned toward Ser 203 at the terminus of the α-helice shown by the purple color in Figure 3, and the A ring of **1** as the C ring of galanthamine located nearby Trp 86. However, the C ring of **1** resided at a different position than the D ring of galanthamine. The C ring of **1** stayed between Tyr 124 and Phe 338, but the D ring of galanthamine was located between Trp 84 (Trp 86 in *Electrophorus electricus*) and Phe 331 (Phe 338 in *Electrophorus electricus*). Once compound **1** entered the substrate binding pocket, it made significant hydrophilic and hydrophobic interactions, including (1) the 3-hydroxyl group acted as the H-bond donator to make contact with Ser 203 and also served as the H-bond acceptor to interact with the backbone of Gly122; (2) the A ring of **1** contacted with Trp 86 and Tyr 337 by π–π interaction; and (3) the C ring of **1** interacted with Tyr 124, Phe 338, and Tyr 341 by π–π interaction.

The substrate binding pocket of acetylcholinesterase mainly includes two important sites, esteric and anionic sites. The esteric site contains some significant hydrophilic residues such as Glu 202 (Glu 199 in *Torpedo californica*), Ser 203 (Ser 200 in *Torpedo californica*), His 447 (His 440 in *Torpedo californica*), and Glu 334 (Glu 327 in *Torpedo californica*), and the potential acetylcholinesterase antagonist should have the structural moiety to make contact with these residues for good inhibition activity. For example, in galanthamine derivatives, whose 9-methoxy group is replaced by a phenoxy or 14-hydroxyl group is replaced by a carbonyl group, their antagonistic effect is largely decreased [31]. For catechin (**1**), galanthamine, and gallic acid (**2**), **1** and galanthamine make similar H-bond interactions, but **2** only makes one H-bond interaction with the backbone of the unimportant residue at the esteric site. Based on these results, the antagonistic effect of **2** should be lower than that of **1** or galanthamine. On the contrary, the anionic site of acetylcholinesterase mainly contains hydrophobic residues such as Trp 86 (Trp 84 in *Torpedo californica*), Tyr 133 (Tyr 130 in *Torpedo californica*), Tyr 341 (Tyr 334 in *Torpedo californica*), Phe 338 (Phe 331 in *Torpedo californica*) and Tyr 449 (Tyr 442 in *Torpedo californica*), so the potential acetylcholinesterase antagonist should have structural moieties to interact with these residues for good inhibition activity. Additionally, the tertiary amino moiety of the potential acetylcholinesterase antagonist located at the anionic site also plays an important role in the antiacetylcholinesterase activity, although it does not exhibit obvious ionic or hydrophilic interaction in the substrate binding pocket. The QSAR result shows that the galanthamine derivative containing a C(3) = N(4) double bond exhibits a better antiacetylcholinesterase effect than that having a C(3)–N(4) single bond. Since the anionic site of acetylcholinesterase contains key aromatic residues, the tertiary amino moiety might interact with the anionic site by the aromatic hydrophobic interaction rather than the ionic interaction. In particular, Trp 86 (Trp 84 in *Torpedo californica*) is the key residue at the anionic site, and it is frequently shown to exhibit a π–π interaction with the acetylcholinesterase antagonist. For example, the crystal structure of the acetylcholinesterase complexed with huperzine A (PDB: 1VOT) from *Torpedo californica* indicates that huperzine A does not make an H-bond interaction with Ser 200 or Glu 199 at the esteric site, and the pyridine moiety of huperzine A makes a strong π–π interaction with Trp 84 [28]. Furthermore, for the binding modes of **1** and galanthamine at the anionic site, the key residue Trp 86 (Trp 84 in *Torpedo californica*), only contacts with the A ring of **1**, but Trp 84 makes three π–π interactions with the C and D rings of galanthamine. According to the evidence mentioned above, it is highly exhibited that the difference in the binding modes of **2**, **1**, and galanthamine at the anionic site might lead to their distinct antiacetylcholinesterase potency.

**Figure 4:**
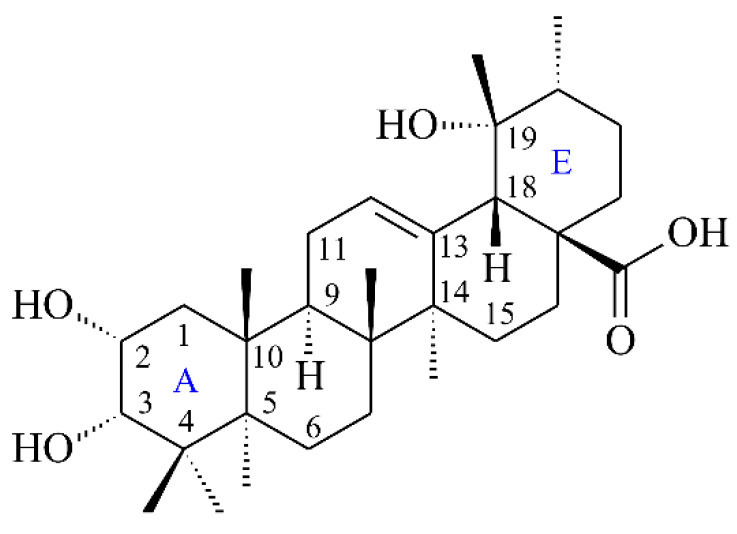
The chemical structure of euscaphic acid (**5**).

**Figure 5:**
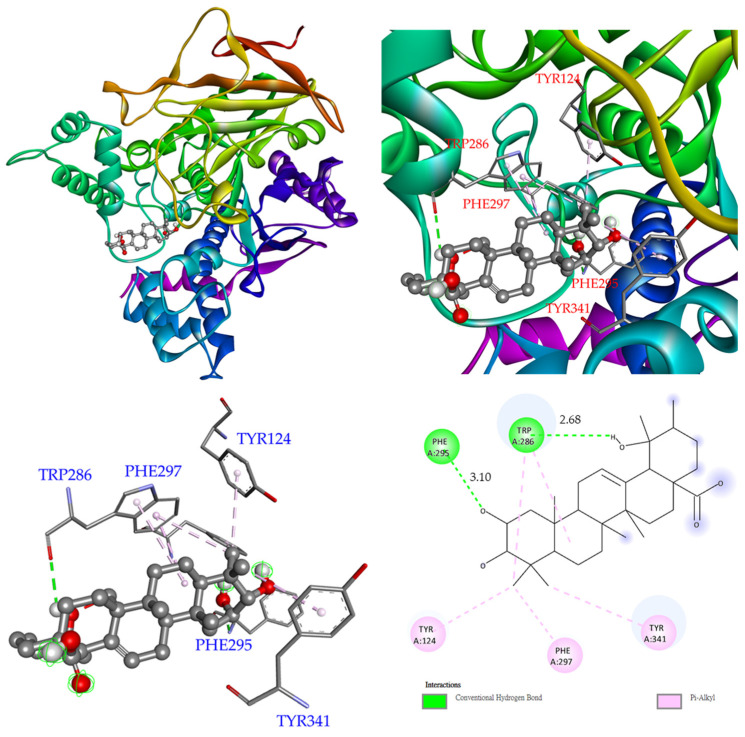
Interactions of euscaphic acid (**5**) with active sites of acetylcholinesterase from *Electrophorus electricus*.

In addition, the interaction between euscaphic acid (**5**) (Figure 4) and acetylcholinesterase was also evaluated. The crystal structure of the acetylcholinesterase from *Electrophorus electricus* (PDB: 1C2B) was employed in this study. For the binding model of **5** (Figure 5), the A ring of **5** stayed between Tyr 124 and Phe 338, a similar position to the C ring of **1**. However, the A ring of **5** resided at a different position from the D ring of galanthamine. The D ring of galanthamine was located between Trp 84 (Trp 86 in *Electrophorus electricus*) and Phe 331 (Phe 338 in *Electrophorus electricus*). **5** made two H-bond interactions comprising (1) the 2-hydroxyl group on the A ring of **5** interacts with Phe 295 by performing as the H-bond acceptor, and (2) the 19-hydroxyl group on the E ring of **5** interacts with Trp 286 by performing as the H-bond acceptor. Additionally, the A ring of **5** interacted with Tyr 124, Tyr 341, and Phe 297 by π–alkyl interaction.

The lowest binding energy of each ligand was regarded as the optimal conformation. In this study, chlorogenic acid and galanthamine were employed as positive control. The binding energies of compounds **5**, **1**, **2**, **3**, **7**, **4**, and **6** were −8.7, −8.5, −8.3, −8.1, −8.1, −7.8, and −7.6 kcal/mol, respectively (Table 7). Compared with chlorogenic acid, the binding energies of compounds **1**–**3**, **5,** and **7** were lower than −8.0 kcal/mol. This shows that **1**–**3**, **5**, and **7** can dock into the pocket of the crystal structure of the acetylcholinesterase from *Electrophorus electricus* more effectively than that of chlorogenic acid.

To further study the interaction between compounds **3**–**5** and α-glucosidase. The crystal structure of the isomaltase from *Saccharomyces cerevisiae* (PDB: 3A4A) was also employed in this study. Compounds **3**–**5** showed potent α-glucosidase inhibitory activity. Therefore, the interaction between **3**–**5** and α-glucosidase was evaluated by molecular model docking. 

In this research, acarbose was employed as a positive control. The binding energies of compounds **3**, **4**, **5**, **6**, **1**, **7**, and **2** were −8.5, −8.0, −7.8, −7.8, −7.6, −7.5, and −5.1 kcal/mol, respectively (Table 8). Compared with the positive control, the binding energies of compounds **1** and **3**–**7** were less than −5.3 kcal/mol. This shows that compounds **1** and **3**–**7** could dock into the pocket of the crystal structure of isomaltase from *Saccharomyces cerevisiae* more effectively than that of acarbose.

**Figure 6:**
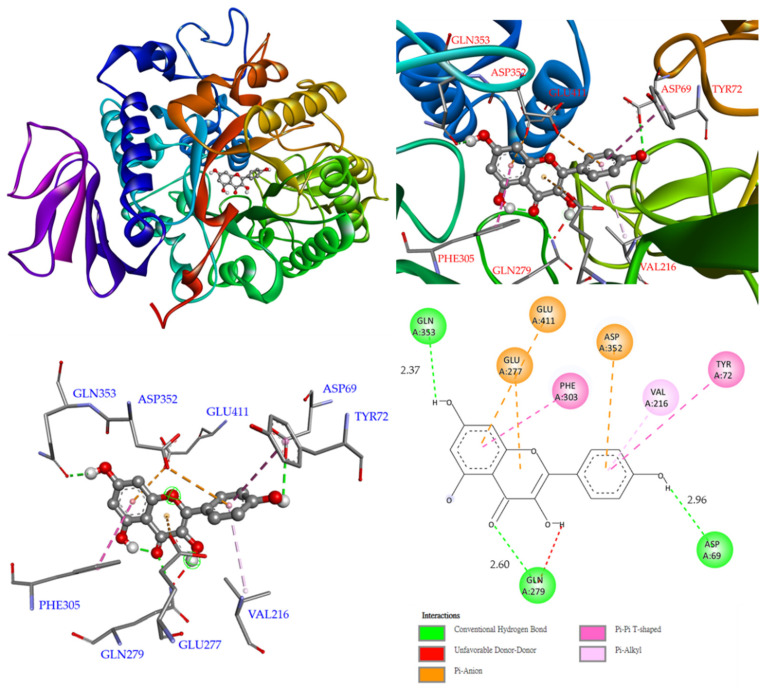
Interactions of kaempferol (**3**) with active sites of *S. cerevisiae* α-glucosidase.

As shown in Figure 6, compound **3** was bound with Gln 353, Gln 277, and Asp 69 through conventional hydrogen bonds, while other interactions (π–π T-shaped, π–alkyl, and π–anion) were also observed with Phe 303, Tyr 72, Val 216, Asp 352, Glu 277, and Glu 411. These permitted **3** and the enzyme to create a stable complex. 

**Figure 7:**
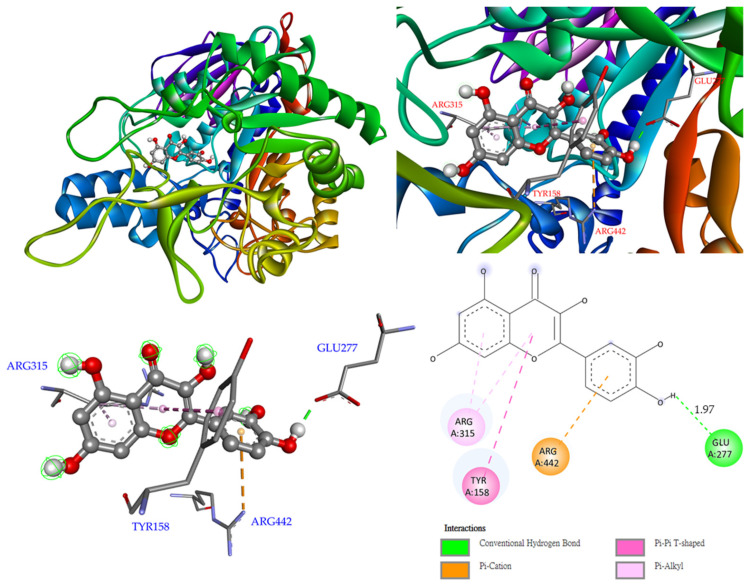
Interactions of quercetin (**4**) with active sites of *S. cerevisiae* α-glucosidase.

For compound **4** (Figure 7), binding to Glu 277 was via conventional hydrogen bonds, while other interactions (π–π T-shaped, π–alkyl, and π–cation) were also observed with Tyr 158, Arg315, and Arg 442. 

**Figure 8:**
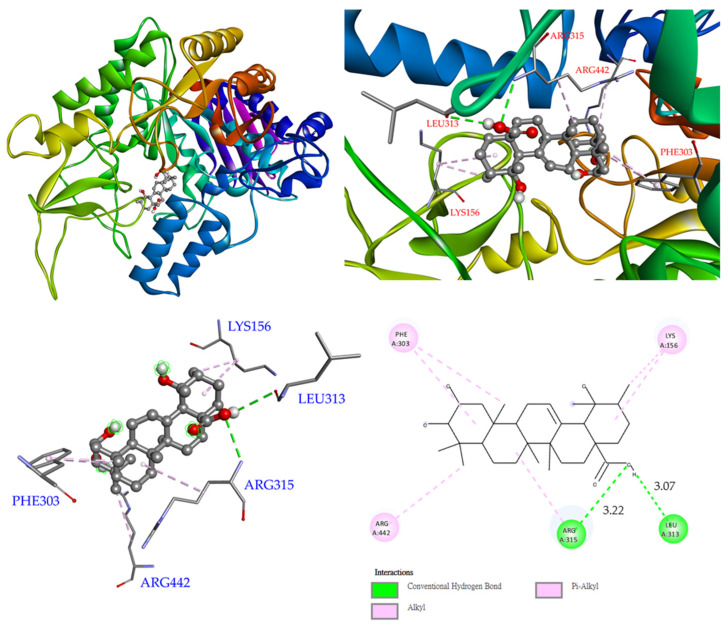
Interactions of euscaphic acid (**5**) with active sites of *S. cerevisiae* α-glucosidase.

Finally, compound **5** was bound with Arg 315 and Leu 313 via conventional hydrogen bonds, while other interactions (alkyl and π–alkyl) were also perceived with Arg 442, Phe 303, and Lys 156 (Figure 8).

**Figure 9:**
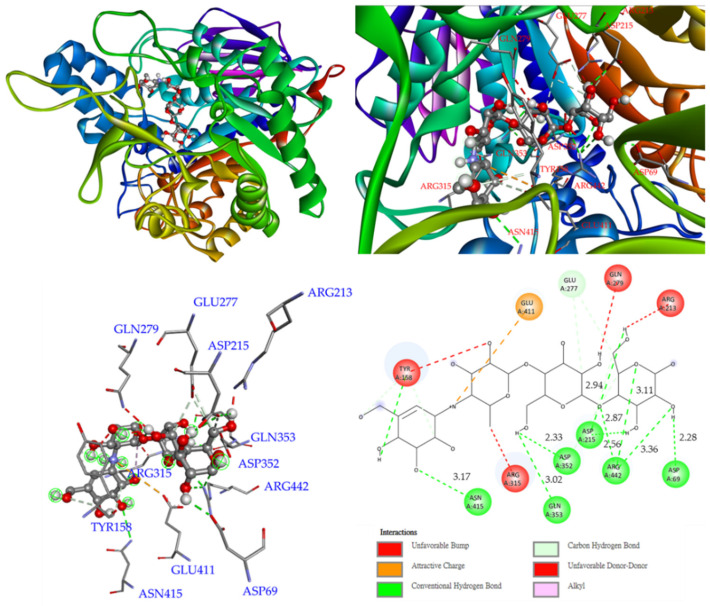
Interactions of acarbose with active sites of *S. cerevisiae* α-glucosidase.

As a positive control, acarbose binds to Arg 442, Asp 69, Asp 215, Asp 352, Asn 415, and Gln 353 via conventional hydrogen bonds. Glu 277 forms a carbon–hydrogen bond with acarbose. Furthermore, two hydroxyl groups provide unfavorable donor–donor interactions with Arg 213 and Gln 279 residues (Figure 9).

On the basis of our data, the docking binding energies of compounds **1** and **3**–**5** are lower than that of acarbose, suggesting that they have better binding capability. In our research, the active ingredients **1** and **3**–**5** not alone exhibited α-glucosidase inhibitory activity but, likewise, had stronger binding potentiality with the active sites of α-glucosidase from *Saccharomyces cerevisiae*. This suggests that these components can be worthy of further research as natural anti-α-glucosidase agents.

## 4. Discussion

Different methods have been used to extract natural products for use as alternatives to modern medicines. Organic solvents play a crucial role in natural product chemistry and are applied to obtain extract products, comprising all kinds of metabolites, based on the property and polarity of the component of interest [32]. Variations in solvent polarity result in dramatic dissimilarities in phytochemical compositions and biological activities. Thus, we used solvents of different polarities to obtain and evaluate these various metabolites from *A. japonica*. In this study, we discovered all kinds of metabolites with different biological activities owing to different solvent polarities. 

In this study, among all solvent extracts evaluated by antioxidant assays such as DPPH, ABTS, and FRAP, acetone extract of *A. japonica* displayed higher antioxidant activity, which may be related to TPC in the extracts. The dissimilarity in antioxidant capacity of various extracts may be due to the different TPC or antioxidant components in each extract. This study is the first report on the comparative evaluation of antioxidant, TFC, and TPC analyses of various solvent extracts from the root of *A. japonica.* This could give a direction for the choice of a suitable solvent for TPC, TFC, and antioxidant extraction methods.

α-Glucosidase has been recognized as a therapeutic target for regulating postprandial hyperglycemia. Inhibition of intestinal α-glucosidase delays carbohydrate digestion and absorption, thereby suppressing postprandial hyperglycemia [33,34]. In the anti-α-glucosidase assay, compounds **1** and **3**–**5** exhibited more potent α-glucosidase inhibitory activities than the positive control acarbose. In this study, it was displayed that **1** was approximately 4-fold stronger than acarbose against α-glucosidase. This study first evaluated the molecular docking study of **5** with *Saccharomyces cerevisiae* α-glucosidase. In addition, the binding energy of **5** to α-glucosidase from *Saccharomyces cerevisiae* was calculated for the first time in our study.

The primary role of acetylcholinesterase is to rapidly hydrolyze acetylcholine at cholinergic synapses, terminating the transmission of nerve impulses. The use of acetylcholinesterase inhibitors to enhance cholinergic function in the brain is a major strategy in the treatment of Alzheimer’s disease (AD) [35]. In previous studies, **1** was used as a pleiotropic drug for the treatment of AD through its cholinesterase inhibitory activity and metal chelating activity. Compound **1** was considered a suitable candidate for the development of neuroprotective agents [36]. In our study, **1**, **2**, and **5** also exhibited anti-acetylcholinesterase activities, which deserves further study. The molecular docking study for **5** with acetylcholinesterase from *Electrophorus electricus* is first assessed in this study. In addition, the binding energy of euscaphic acid to *Electrophorus electricus* acetylcholinesterase was calculated for the first time in our study.

## 5. Conclusions

Different solvent extracts of *A. japonica* were studied by anti-acetylcholinesterase, anti-α-glucosidase, and antioxidant assays. The contents of TPC in methanol and ethanol extracts were about 5 to 6 times those of TPC in chloroform, dichloromethane and *n*-hexane extracts, which proved that the suitable relative polarity range of TPC in *A. japonica* extraction solvents was 0.355 to 0.762. The low-polarity solvent extracts, like chloroform and ethyl acetate, had higher amount of TFC than high-polarity solvent extracts. The TFC in the chloroform extract was almost 20-fold that of the water extract. In our study, acetone, ethanol, and methanol extracts displayed relatively strong ABTS, DPPH, superoxide radical scavenging, and FRAP activities, which may be consistent with TPC in the extracts. The acetone extract had the highest antioxidant and anti-α-glucosidase activities. For the AChE inhibitory activities, the ethanol, methanol and acetone extract of *A. japonica* showed higher inhibitory effects than other solvent extracts. Biological activity assays showed that compounds **1**–**4** and **6**–**7** displayed antioxidant activities, and compounds **1** and **3**–**7** had strong anti-α-glucosidase effects. Compound **1**–**5** and **7** all showed good AChE inhibitory activity. As the result of molecular docking, **1** and **5** at the anionic site might lead to its distinct anti-acetylcholinesterase potency. On the other hand, compounds **3**–**5** had better binding capacity to the active site of α-glucosidase from *Saccharomyces cerevisiae*. 

In summary, this study revealed that the extraction solvent of *A. japonica* affected the extraction yield, antioxidant effect and other biological activities. The acetone, methanol, and ethanol extracts showed relatively high TPC levels and antioxidant activities. The acetone extract contains the most polyphenols, and the chloroform extract contains the most flavonoids. In addition, the ethanol, acetone and methanol extracts showed higher inhibitory effects against α-glucosidase and AChE. According to our antioxidant results, the active antioxidant components of *A. japonica* were compounds **1**–**4** and **6**–**7**. The above bioactive components could be applied as herbal antioxidants against oxidative damage. It is also worth noting that they also act as natural α-glucosidase and acetylcholinesterase inhibitors.

## Figures and Tables

**Table 1:** Each extraction solvent from *Ampelopsis japonica* of TPC, TFC, and extraction yields.

Extracting Solvents	Relative Polarity	TPC (mg/g) ^a^ (GAE)	TFC (mg/g) ^b^ (QE)	Yields (%) ^c^
*n*-Hexane	0.009	15.01 ± 0.44 ***	56.36 ± 3.86 **	0.28 ± 0.08
Chloroform	0.259	20.92 ± 1.47 **	94.22 ± 1.34 ***	0.50 ± 0.08
Dichloromethane	0.269	20.19 ± 1.99 **	62.60 ± 3.51 ***	0.45 ± 0.04
Ethyl acetate	0.228	79.09 ± 8.45 **	80.05 ± 7.82 **	0.66 ± 0.10
Acetone	0.355	142.89 ± 4.07 ***	71.72 ± 2.66 ***	1.03 ± 0.27
Methanol	0.762	95.69 ± 7.80 **	5.72 ± 1.13 **	1.90 ± 0.50
Ethanol	0.654	95.98 ± 6.68 **	22.75 ± 1.24 ***	4.87 ± 0.03
Water	1.000	47.38 ± 2.18 ***	4.45 ± 1.21 *	16.46 ± 0.31

^a^ TPC was shown as milligrams of gallic acid equivalents (GAE) per gram of extract. ^b^ TFC was shown as milligrams of quercetin equivalents (QE) per gram of extract; ^c^ Yield was counted as % yield = (weight of extract/initial weight of dry *Ampelopsis japonica*) × 100; * *p* < 0.05; ** *p* < 0.01; *** *p* < 0.001 compared with the control.

**Table 2:** Antioxidant effects of various solvent extracts from *Ampelopsis japonica* determined by DPPH, ABTS, superoxide radical scavenging, and FRAP assays.

Extracting Solvents	SC_50_ (μg/mL) ^a^			TE (mM/g) ^d^
	DPPH	ABTS	Superoxide	FRAP
*n*-Hexane	>200	>200	>400	26.03 ± 1.90 **
Chloroform	>200	>200	>400	94.07 ± 7.35 ***
Dichloromethane	>200	>200	>400	104.20 ± 9.18 ***
Ethyl acetate	92.14 ± 8.12 *	57.45 ± 4.74 *	>400	587.11 ± 20.61 ***
Acetone	54.88 ± 4.40 *	33.88 ± 2.31 **	>400	1001.00 ± 46.17 ***
Methanol	84.73 ± 7.82 *	53.77 ± 4.65 **	290.83 ± 15.23 *	712.56 ± 18.32 ***
Ethanol	87.12 ± 6.45 *	64.56 ± 4.80 **	307.20 ± 22.39 *	736.95 ± 14.40 ***
Water	98.54 ± 7.09 *	99.30 ± 7.02 *	313.84 ± 20.24 *	413.34 ± 21.08 ***
BHT ^b^	33.04 ± 2.12 **	14.09 ± 0.24 **	N.A. ^c^	4257.97 ± 145.90 ***

^a^ The SC_50_ value was defined as the concentration of the samples causing 50% free radical scavenging, and was displayed as mean ± SD (n = 3); ^b^ BHT was employed as positive control; ^c^ N.A. means unavailable (poor solubility); ^d^ FRAP was expressed in millimolar of Trolox equivalents (TE) per gram of extract; * *p* < 0.05, ** *p* < 0.01, and *** *p* < 0.001 compared with the control.

**Table 3:** Different solvent extracts from *Ampelopsis japonica* of α-glucosidase and AChE inhibitory activities.

Extracting Solvents	IC_50_ (μg/mL) ^a^	
	α-Glucosidase	AChE
*n*-Hexane	28.43 ± 3.78 *	83.97 ± 8.90 *
Chloroform	34.16 ± 3.88 *	91.64 ± 8.77 *
Dichloromethane	28.00 ± 0.14 *	91.47 ± 26.03 *
Ethyl acetate	12.51 ± 2.42 *	103.30 ± 2.15 *
Acetone	8.30 ± 0.78 **	61.95 ± 5.54 **
Methanol	19.27 ± 1.12 *	77.99 ± 5.08 *
Ethanol	11.06 ± 2.07 *	37.08 ± 7.67 *
Water	>400	85.82 ± 8.74 *
Acarbose ^b^	335.50 ± 2.14 *	—
Chlorogenic acid ^b^	—	66.69 ± 0.16 *

^a^ The IC_50_ value was defined as half-maximal inhibitory concentration of each free radical scavenging activity; BHT was employed as positive control; ^b^ Acarbose and chlorogenic acid were employed as positive controls; * *p* < 0.05 and ** *p* < 0.01 compared with the control.

**Table 4:** Quantification and identification of the main active compounds from *Ampelopsis japonica* in various extracts.

Extracting Solvents	mg/kg							
	1	2	3	4	5	6	7	Total Amount
Water	6.27 ± 0.58	8.43 ± 0.73	4.64 ± 0.22	22.30 ± 1.62	0.68 ± 0.03	N.D.	N.D.	42.32 ± 3.18
Methanol	10.27 ± 1.08	12.73 ± 1.42	7.24 ± 0.64	9.30 ± 0.91	1.26 ± 0.08	3.01 ± 0.28	0.67 ± 0.03	44.48 ± 4.44
Ethanol	7.85 ± 0.52	16.43 ± 1.64	2.94 ± 0.03	3.71 ± 0.28	0.86 ± 0.07	11.22 ± 1.02	0.58 ± 0.04	43.59 ± 3.60
Acetone	8.43 ± 0.63	12.43 ± 1.72	3.21 ± 0.06	6.64 ± 0.74	1.21 ± 0.02	12.42 ± 1.44	3.41 ± 0.18	47.75 ± 4.79
Ethyl acetate	3.79 ± 0.16	8.86 ± 0.63	1.54 ± 0.08	3.62 ± 0.21	1.01 ± 0.08	2.60 ± 0.11	0.52 ± 0.09	21.94 ± 1.36
Chloroform	4.12 ± 0.34	2.63 ± 0.08	7.83 ± 0.55	4.02 ± 0.43	0.91 ± 0.06	0.70 ± 0.08	0.84 ± 0.07	21.05 ± 1.61
Dichloromethane	2.74 ± 0.16	1.63 ± 0.06	4.62 ± 0.43	2.66 ± 0.12	0.84 ± 0.08	1.83 ± 0.06	0.71 ± 0.07	15.03 ± 0.98
*n*-Hexane	1.43 ± 0.08	3.46 ± 0.26	1.12 ± 0.07	2.63 ± 0.22	0.69 ± 0.07	6.23 ± 0.52	N.D.	15.56 ± 1.22

Results are expressed as milligrams of each compound in kilograms of extract. N.D. means no detectable.

**Table 5:** Antioxidant effects of isolated compounds from *Ampelopsis japonica* measured with DPPH, ABTS, superoxide radical scavenging, and FRAP assays.

Compounds	SC_50_ (μg/mL) ^a^			TE (mM/g)
	DPPH	ABTS	Superoxide	FRAP
**1**	10.08 ± 3.09 **	2.23 ± 0.22 **	64.43 ± 7.73 *	8729.33 ± 424.55 ***
**2**	2.60 ± 0.67 *	1.45 ± 0.14 **	47.40 ± 3.01 *	28,512.82 ± 43.27 ***
**3**	12.48 ± 3.01 **	5.24 ± 0.45 *	N.A. ^−b^	7912.47 ± 220.08 ***
**4**	3.36 ± 0.58 **	3.15 ± 0.49 *	31.89 ± 2.03 **	16,038.26 ± 86.89 ***
**5**	>400	>400	>400	8.67 ± 3.93 *
**6**	13.19 ± 4.78 *	2.81 ± 0.12 **	66.16 ± 5.23 *	7453.94 ± 60.09 ***
**7**	2.78 ± 0.25 *	3.78 ± 0.03 **	41.76 ± 4.20 *	13,122.77 ± 182.42 ***
BHT ^b^	36.99 ± 4.54 *	17.36 ± 3.14 *	N.A. ^c^	3997.23 ± 144.35 ***

^a^ The SC_50_ value was defined as the concentration of the samples causing 50% free radical scavenging, and was displayed as mean ± SD (n = 3); ^b^ BHT was employed as positive control; ^c^ N.A. means unavailable (poor solubility); * *p* < 0.05, ** *p* < 0.01, and *** *p* < 0.001 compared with the control.

**Table 6:** Main isolated components from *Ampelopsis japonica* of α-glucosidase and AChE inhibitory activities.

Compounds	IC_50_ (μg/mL)	
	α-Glucosidase	AChE
**1**	81.78 ± 11.58 **	26.35 ± 9.55 **
**2**	>400	41.59 ± 7.57 *
**3**	5.81 ± 2.70 **	55.04 ± 8.57 **
**4**	14.39 ± 5.93 **	66.34 ± 5.09 **
**5**	20.38 ± 2.13 *	11.64 ± 2.69 **
**6**	28.81 ± 5.65 *	80.75 ± 9.21 **
**7**	88.73 ± 10.94 *	53.38 ± 7.30 *
Acarbose ^a^	334.53 ± 2.22 *	—
Chlorogenic acid ^a^	—	64.42 ± 0.16 *
Galanthamine hydrobromide ^a^	—	0.57 ± 0.09 *

^a^ Acarbose, chlorogenic acid and galanthamine hydrobromide were employed as positive controls; * *p* < 0.05 and ** *p* < 0.01 compared with the control.

**Table 7:** Binding energies of active components with acetylcholinesterase from *Electrophorus electricus* calculated in silico.

Compounds	Affinity (kcal/mol)
**1**	−8.5
**2**	−8.3
**3**	−8.1
**4**	−7.8
**5**	−8.7
**6**	−7.6
**7**	−8.1
Chlorogenic acid ^a^	−8.0
Galanthamine ^a^	−9.4

^a^ Chlorogenic acid and galanthamine employed as positive controls.

**Table 8:** Binding energies of active components and acarbose with α-glucosidase from *Saccharomyces cerevisiae* calculated in silico.

Compounds	Affinity (kcal/mol)
**1**	−7.6
**2**	−5.1
**3**	−8.5
**4**	−8.0
**5**	−7.8
**6**	−7.8
**7**	−7.5
Acarbose ^a^	−5.3

^a^ Acarbose employed as a positive control.

## Data Availability

The data presented in this study are available in the main text and the supplementary materials of this article.

## Supplementary Materials

The following supporting information can be downloaded at: https://www.mdpi.com/article/10.3390/antiox11071228/s1. Table S1: Retention time, LODs, LOQs, and regression analysis for seven components in *Ampelopsis japonica* in reverse phase. Figure S1: ^1^H-NMR spectrum (acetone-*d*_6_, 400 MHz) of catechin (**1**). Figure S2: ^1^H-NMR spectrum (acetone-*d_6_*, 400 MHz) of gallic acid (**2**). Figure S3: ^1^H-NMR spectrum (acetone-*d_6_*, 400 MHz) of kaempferol (**3**). Figure S4: ^1^H-NMR spectrum (acetone-*d*_6_, 400 MHz) of quercetin (**4**). Figure S5: ^1^H-NMR spectrum (methanol-*d*_4_, 500 MHz) of euscaphic acid (**5**). Figure S6: ^1^H-NMR spectrum (acetone-*d*_6_, 400 MHz) of resveratrol (**6**). Figure S7: ^1^H-NMR spectrum (methanol-*d*_4_, 400 MHz) of epicatechin (**7**). Figure S8: Reversed-phase HPLC chromatogram of isolated pure compounds. Figure S9: Reversed-phase HPLC chromatogram of water extract. Figure S10: Reversed-phase HPLC chromatogram of methanol extract. Figure S11: Reversed-phase HPLC chromatogram of ethanol extract. Figure S12: Reversed-phase HPLC chromatogram of acetone extract. Figure S13: Reversed-phase HPLC chromatogram of ethyl acetate extract. Figure S14: Reversed-phase HPLC chromatogram of dichloromethane extract. Figure S15: Reversed-phase HPLC chromatogram of chloroform extract. Figure S16: Reversed-phase HPLC chromatogram of *n*-hexane extract. Figure S17: Reversed-phase HPLC chromatogram of all solvent extracts with the same scale.
