# Supplementary material for: Bioactive Components from Ampelopsis japonica with Antioxidant, Anti-α-Glucosidase, and Antiacetylcholinesterase Activities
Source: Antioxidants (Basel). 2022 Jun 23;11(7):1228. doi: 10.3390/antiox11071228 (PMC9312113; doi:10.3390/antiox11071228)
Supplement: Supplementary file 1 [file antioxidants-11-01228-s001.zip › antioxidants-1755392-supplementary.pdf]

Supplementary data

Bioactive Components from *Ampelopsis japonica* with Antioxidant, Anti-α-Glucosidase, and Anti-Acetylcholinesterase Activities

Jia-Hua Liang [1], Hsiang-Ru Lin [2], Chang-Syun Yang [3], Chia-Ching Liaw [4,5], I-Chou Wang [6,7,*] and Jih-Jung Chen [3,8,*]

[1] Institute of Traditional Medicine, School of Medicine, National Yang Ming Chiao Tung University, Taipei 112304, Taiwan; liangjia.ps08@nycu.edu.tw (J.-H.L.)
[2] Department of Chemistry, College of Science, National Kaohsiung Normal University, Kaohsiung 82444, Taiwan; t3136@nknu.edu.tw (H.-R.L.)
[3] Department of Pharmacy, School of Pharmaceutical Sciences, National Yang Ming Chiao Tung University, Taipei 112304, Taiwan; tim0619@nycu.edu.tw (C.-S.Y.)
[4] National Research Institute of Chinese Medicine, Ministry of Health and Welfare, Taipei 112026, Taiwan; liawcc@nricm.edu.tw (C.-C.L.)
[5] Department of Biochemical Science and Technology, National Chiayi University, Chiayi 600355, Taiwan
[6] Department of Physical Medicine and Rehabilitation, Kaohsiung Veterans General Hospital Tainan Branch, Tainan City 710011, Taiwan; bm840000@mail.vhyk.gov.tw
[7] Department of Food Nutrition, Chung Hwa University of Medical Technology, Tainan City 717302, Taiwan
[8] Department of Medical Research, China Medical University Hospital, China Medical University, Taichung 404332, Taiwan
* Correspondence: bm840000@mail.vhyk.gov.tw (I.-C.W.); jjungchen@nycu.edu.tw (J.-J.C.); Tel.: +886-2-2826-7195 (J.-J.C.); Fax: +886-2-2823-2940 (J.-J.C.)

Contents

Table S1. Retention time, LODs, LOQs, and regression analysis for seven components of *Ampelopsis japonica* in reverse phase HPLC....................S3

Figure S1. ^{1}H-NMR spectrum (acetone-d_6, 400 MHz) of catechin (**1**) S4

Figure S2. ^{1}H-NMR spectrum (acetone-d_6, 400 MHz) of gallic acid (**2**) S4

Figure S3. ^{1}H-NMR spectrum (acetone-d_6, 400 MHz) of kaempferol (**3**) S5

Figure S4. ^{1}H-NMR spectrum (acetone-d_6, 400 MHz) of quercetin (**4**) S5

Figure S5. ^{1}H-NMR spectrum (methanol-d_4, 500 MHz) of euscaphic acid (**5**) S6

Figure S6. ^{1}H-NMR spectrum (acetone-d_6, 400 MHz) of resveratrol (**6**) S7

Figure S7. ^{1}H-NMR spectrum (methanol-d_4, 400 MHz) of epicatechin (**7**) S7

Figure S8. Reverse-phase HPLC chromatogram of isolated compounds....................S8

Figure S9. Reverse-phase HPLC chromatogram of water extract…....................S9

Figure S10. Reverse-phase HPLC chromatogram of methanol extract....................S9

Figure S11. Reverse-phase HPLC chromatogram of ethanol extract....................S9

Figure S12. Reverse-phase HPLC chromatogram of acetone extract....................S10

Figure S13. Reverse-phase HPLC chromatogram of ethyl acetate extract....................S10

Figure S14. Reverse-phase HPLC chromatogram of dichloromethane extract....................S10

Figure S15. Reverse-phase HPLC chromatogram of chloroform extract…....................S11

Figure S16. Reverse-phase HPLC chromatogram of *n*-hexane extract……....................S11

Figure S17. Reverse-phase HPLC chromatogram of all solvent extracts with the same scale ...S12

Table S1. Retention time, LODs, LOQs, and regression analysis for seven components of *Ampelopsis japonica* in reverse phase HPLC.

Compounds	T_m (min) [a]	Regression equation	Correlation coefficient	LOD (μg/mL) [a]	LOQ (μg/mL) [a]
Resveratrol	68.0	y = 24996x-5421.3	0.9997	5.75	17.44
Gallic acid	80.0	y = 20108x+3245.3	0.9993	9.62	29.16
Catechin	90.0	y = 12165x-2898.5	0.9996	14.75	44.70
Quercetin	95.0	y = 27262x + 957.4	0.9998	7.78	23.57
Kaempferol	106.0	y = 29758x +1079.9	0.9997	5.35	16.22
Epicatechin	110.0	y = 32979x+5882.9	0.9999	6.24	18.90
Euscaphic acid	126.0	y = 13430x − 474.4	0.9995	13.42	40.68

[a] T_m: Retention time; LOD: Limit of detection; LOQ: Limit of quantification; LOD was counted as = 3.3 × the standard deviation of the intercept / the slope of the standard curve; LOQ was counted as = 10 × the standard deviation of the intercept / the slope of the standard curve. (LOD and LOQ was calculated with reference to the International Conference on Harmonization's (ICH) content.)

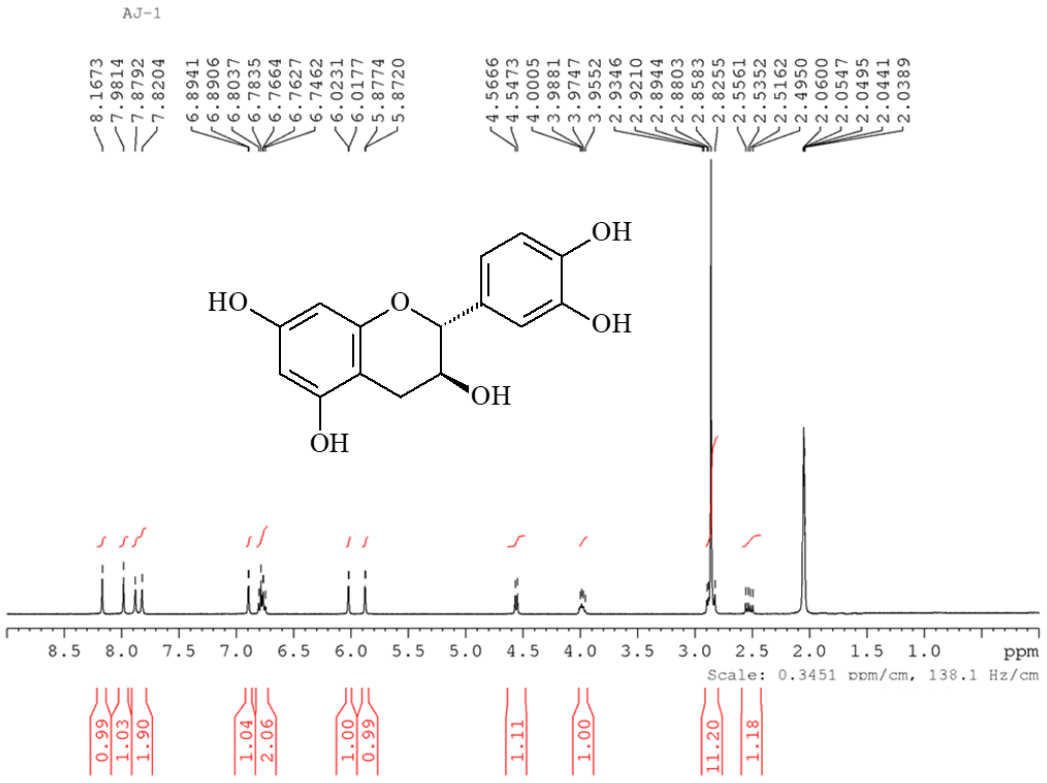

Figure S1. ^{1}H-NMR spectrum (acetone-d_6, 400 MHz) of catechin (**1**).

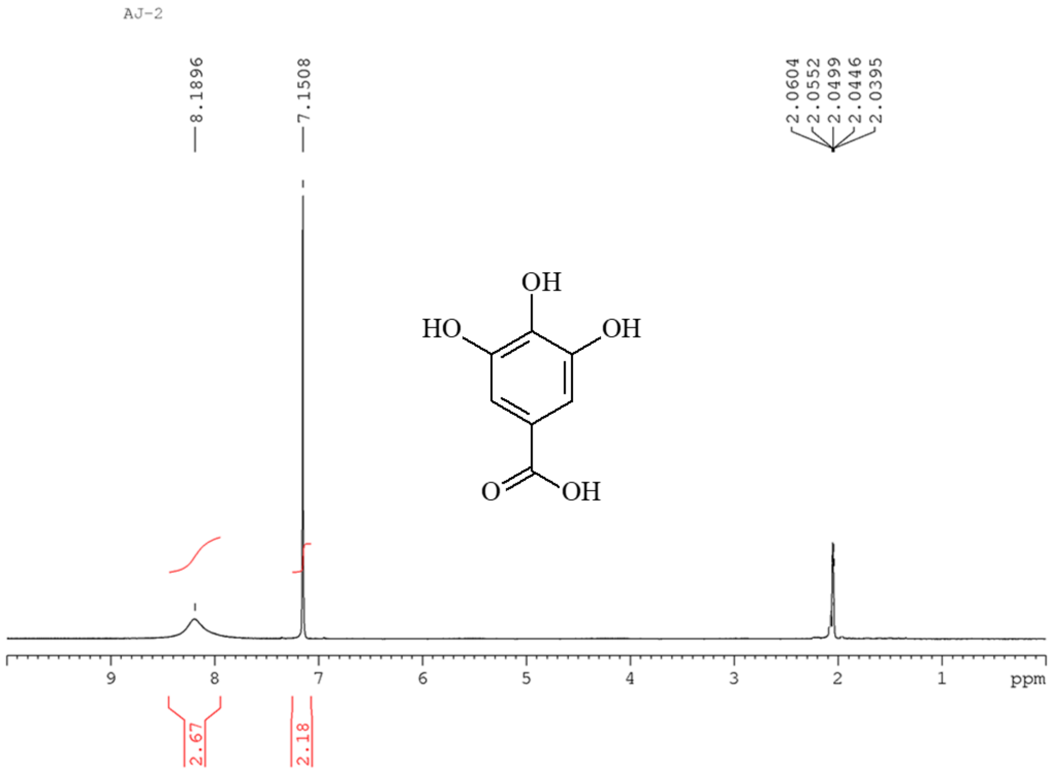

Figure S2. ^{1}H-NMR spectrum (acetone-d_6, 400 MHz) of gallic acid (**2**).

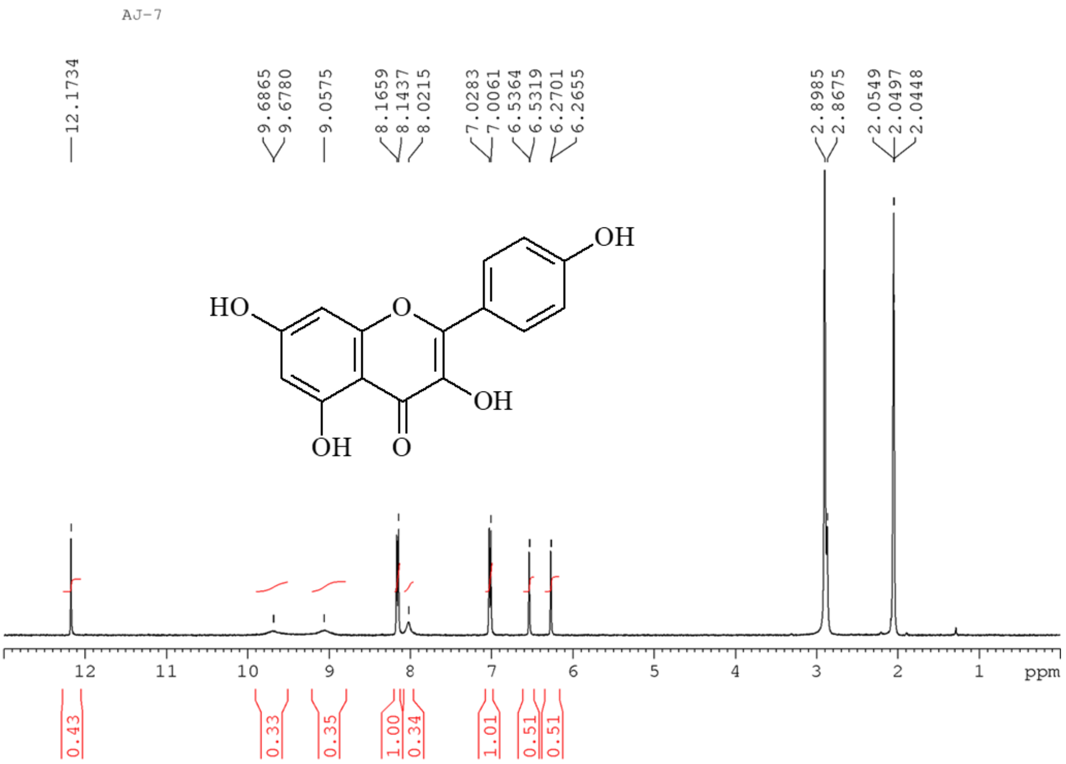

Figure S3. ^{1}H-NMR spectrum (acetone-d_6, 400 MHz) of kaempferol (**3**).

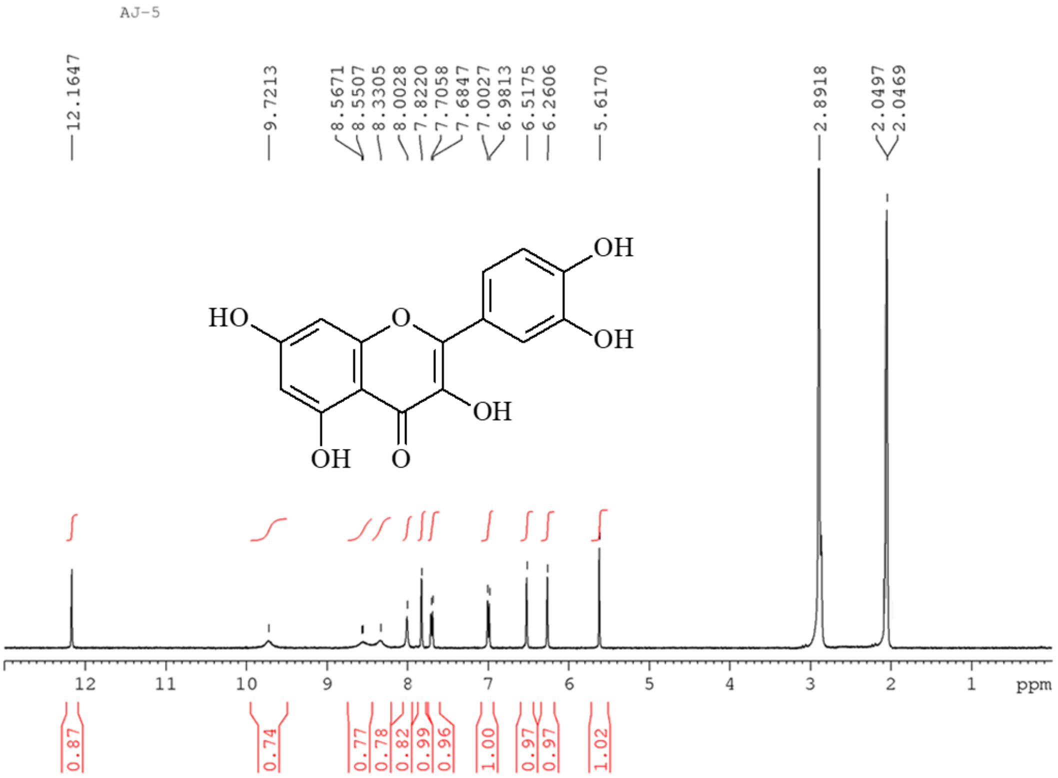

Figure S4. ^{1}H-NMR spectrum (acetone-d_6, 400 MHz) of quercetin (**4**).

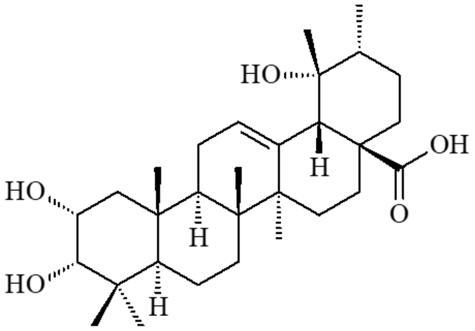

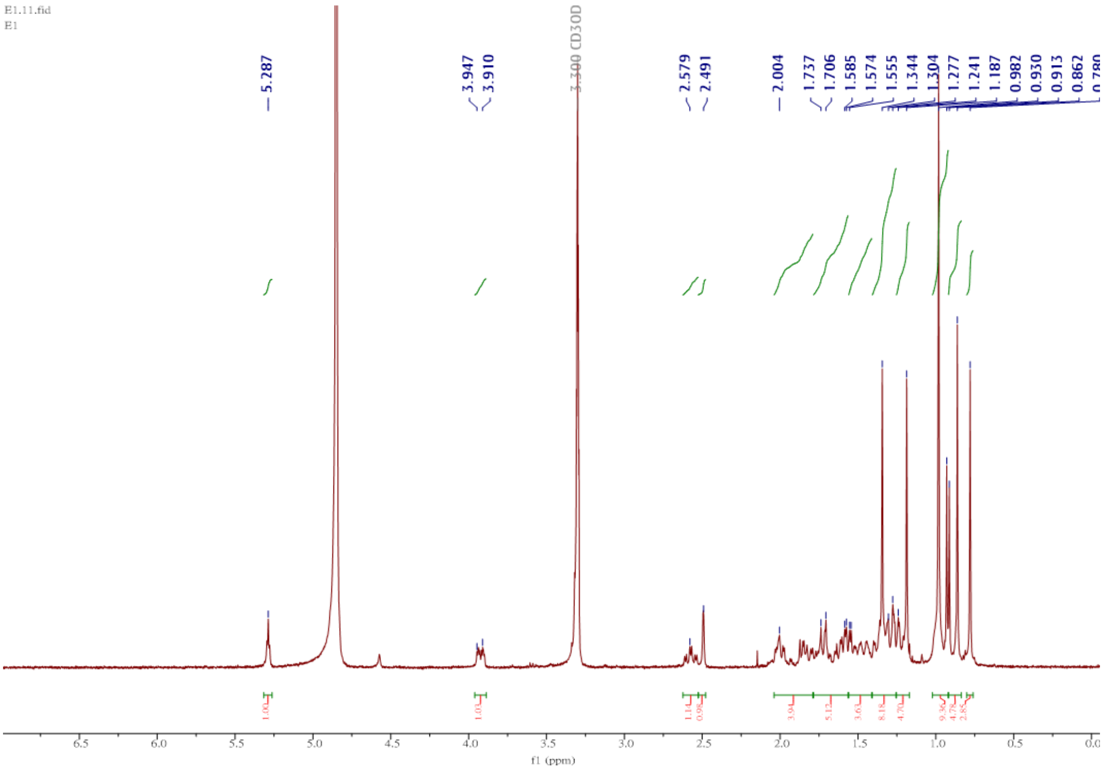

Figure S5. ^{1}H-NMR spectrum(methanol-d_4, 500 MHz) of euscaphic acid (**5**).

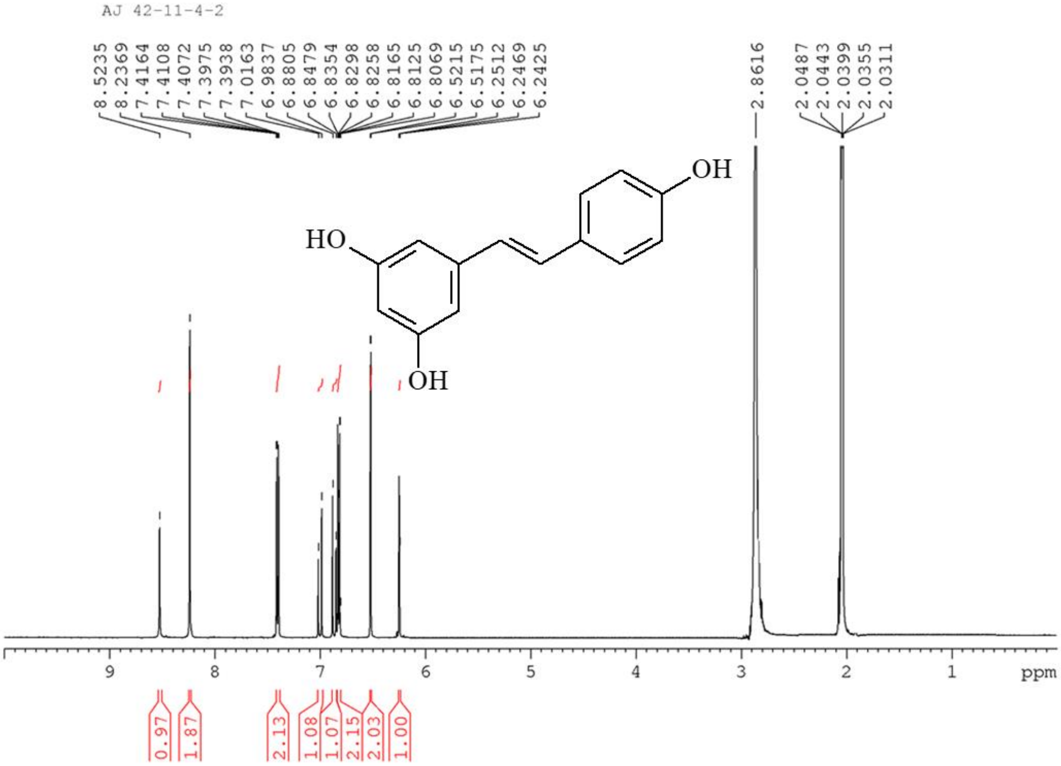

Figure S6. ^{1}H-NMR spectrum(acetone-d_6, 400 MHz) of resveratrol (**6**).

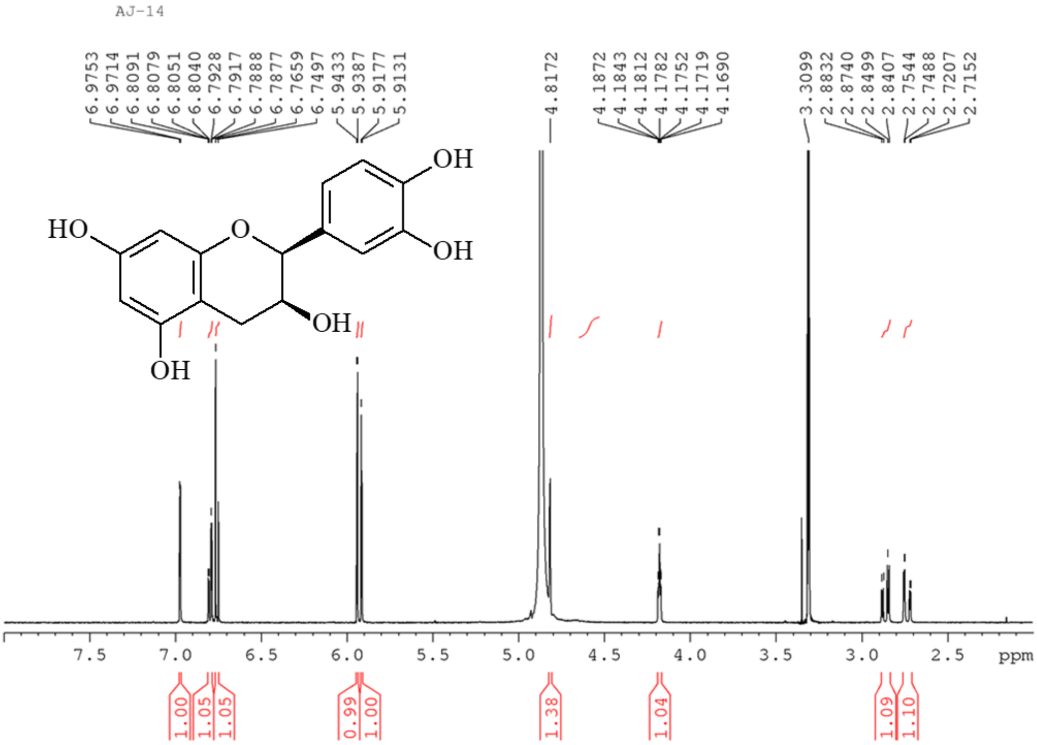

Figure S7. ^{1}H-NMR spectrum(methanol-d_4, 400 MHz) of epicatechin (**7**).

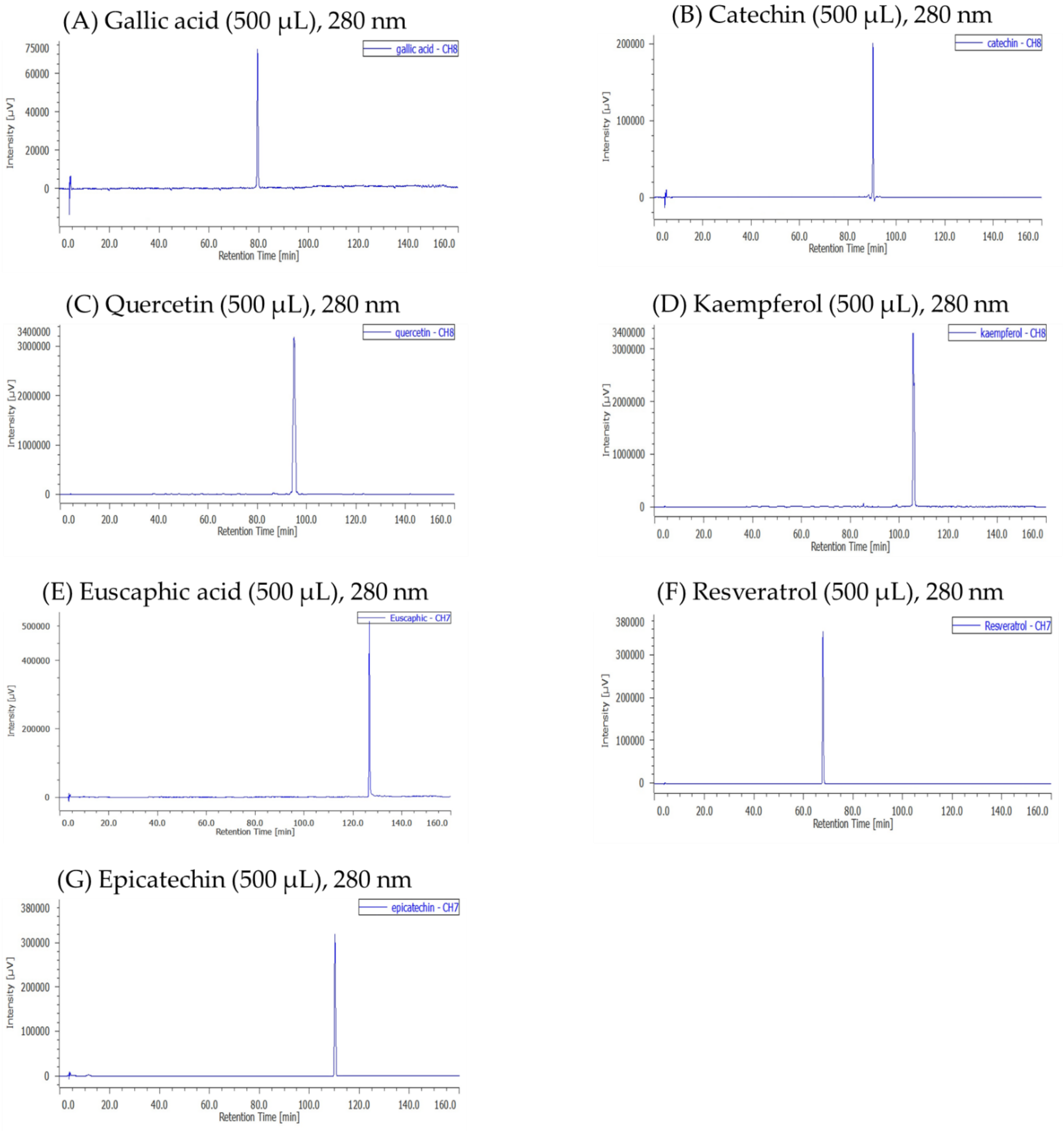

Figure S8. Reverse-phase HPLC chromatogram of isolated compounds (A to G).

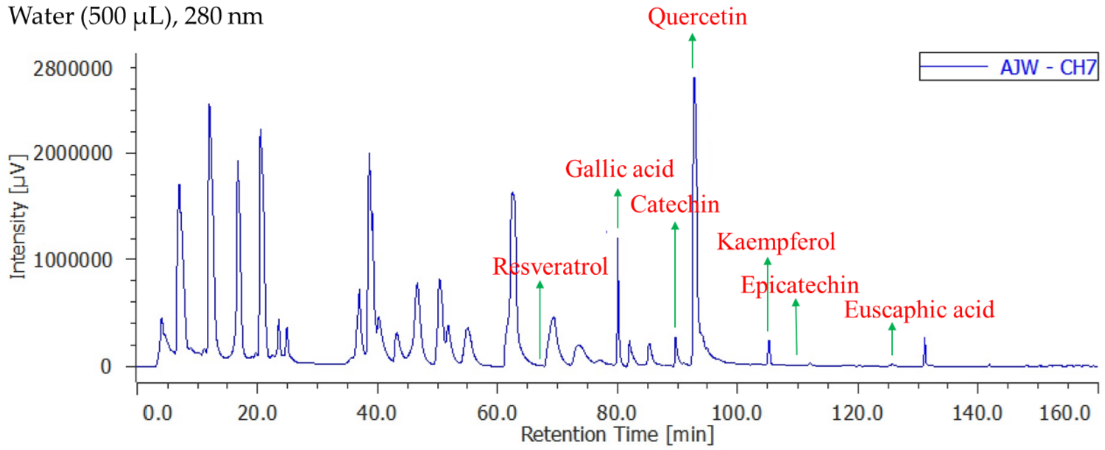

Figure S9. Reverse-phase HPLC chromatogram of water extract.

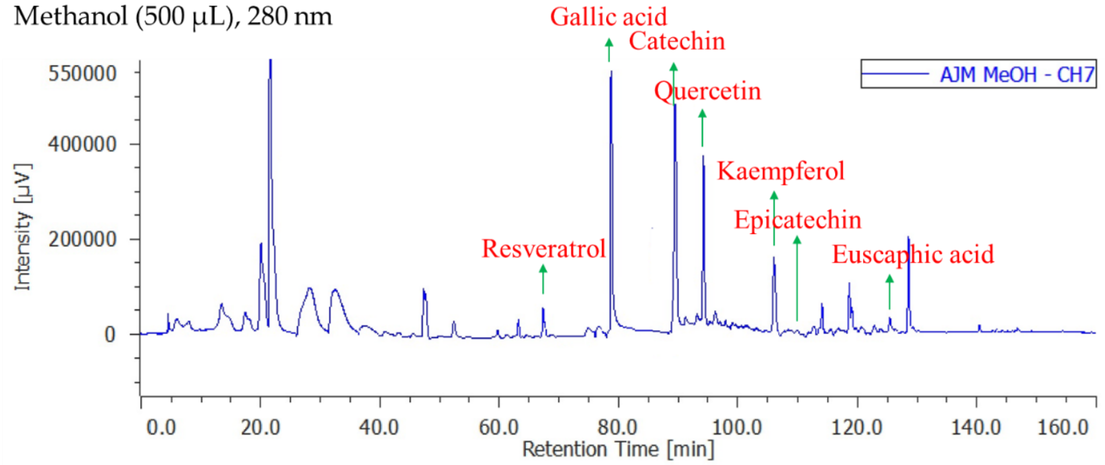

Figure S10. Reverse-phase HPLC chromatogram of methanol extract.

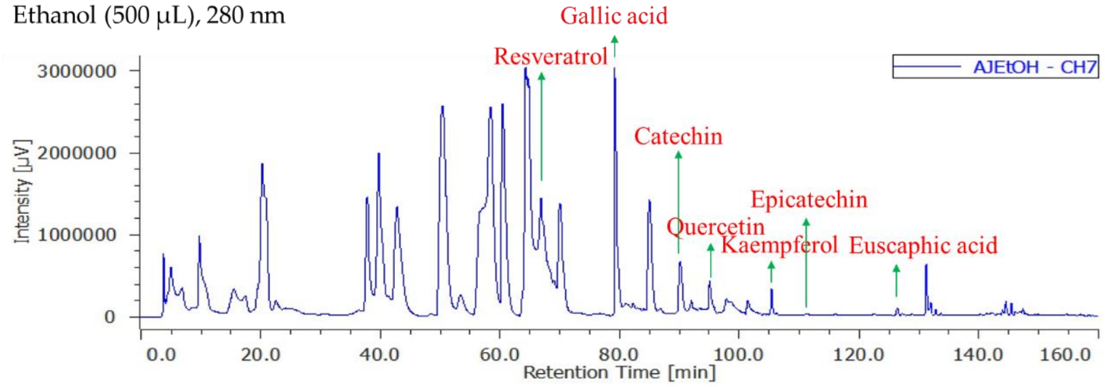

Figure S11. Reverse-phase HPLC chromatogram of ethanol extract.

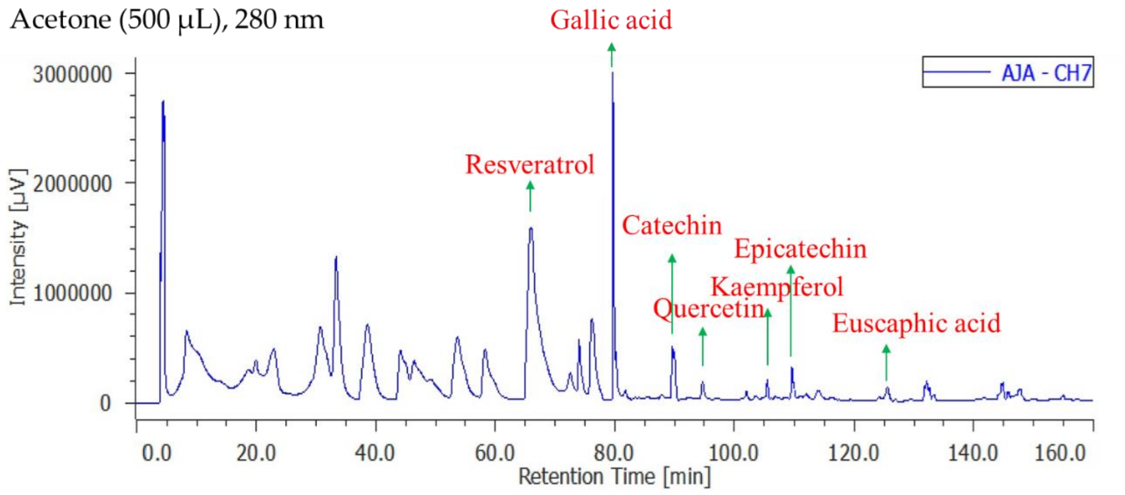

Figure S12. Reverse-phase HPLC chromatogram of acetone extract.

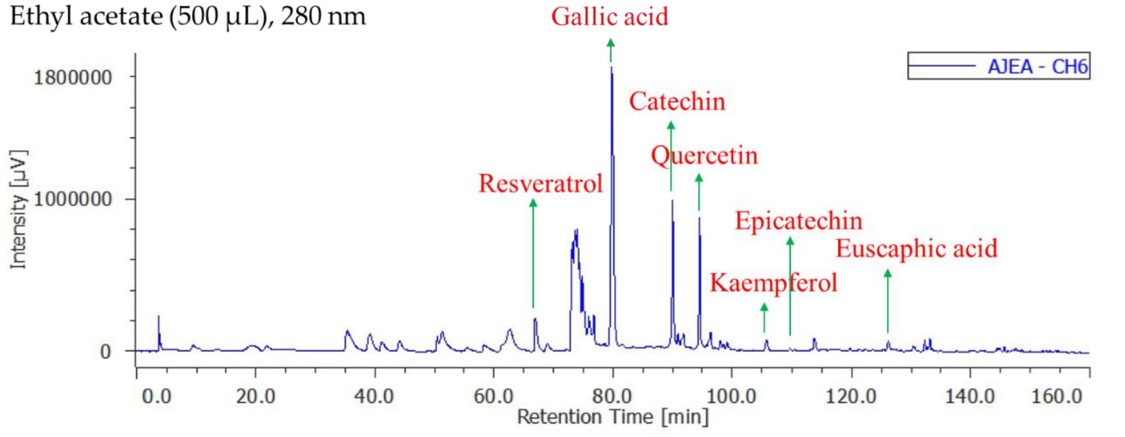

Figure S13. Reverse-phase HPLC chromatogram of ethyl acetate extract.

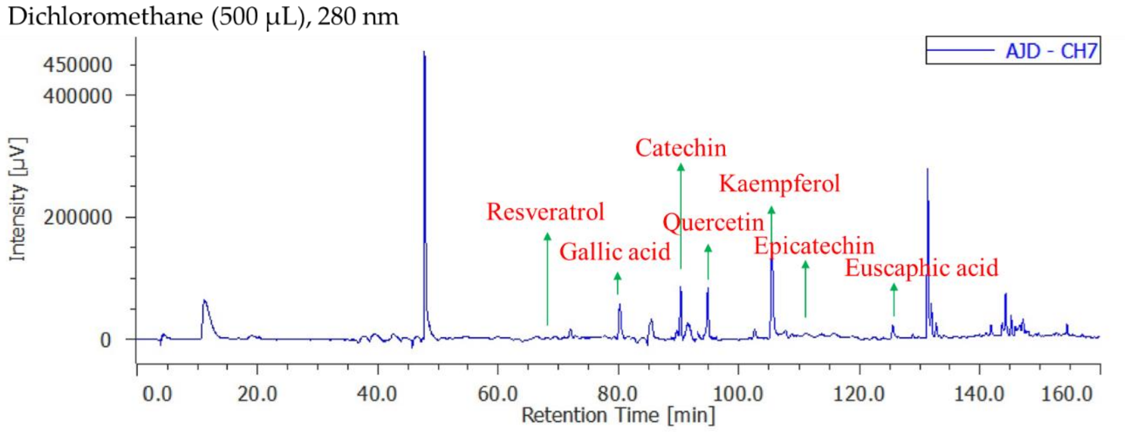

Figure S14. Reverse-phase HPLC chromatogram of dichloromethane extract.

Chloroform (500 µL), 280 nm

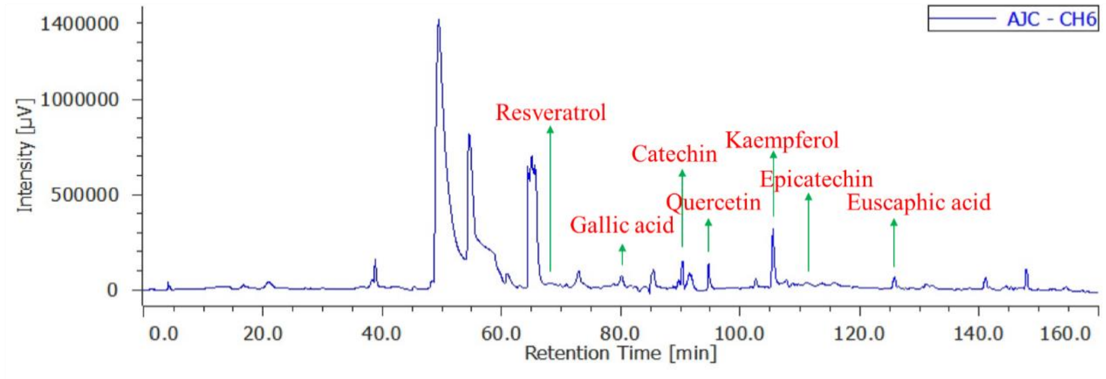

Figure S15. Reverse-phase HPLC chromatogram of chloroform extract.

n-Hexane (500 µL), 280 nm

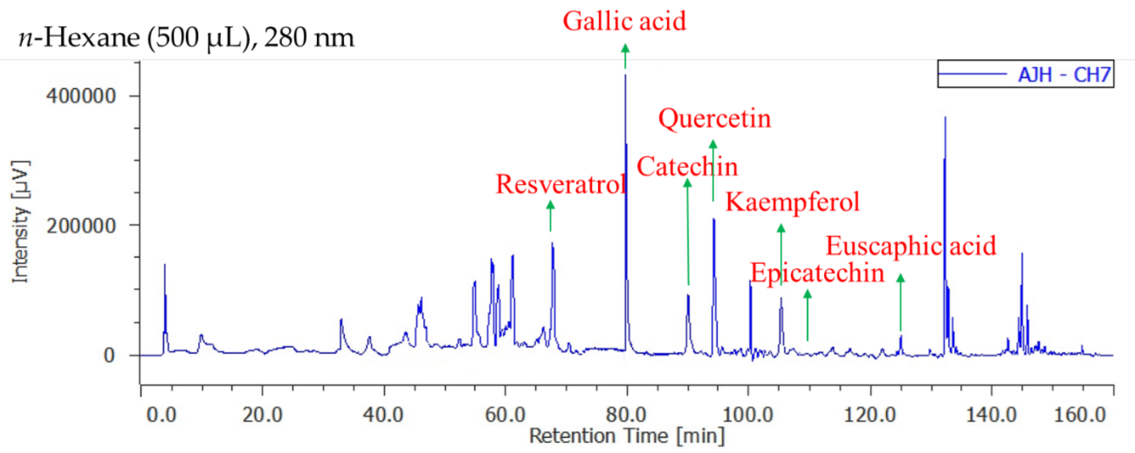

Figure S16. Reverse-phase HPLC chromatogram of *n*-hexane extract.

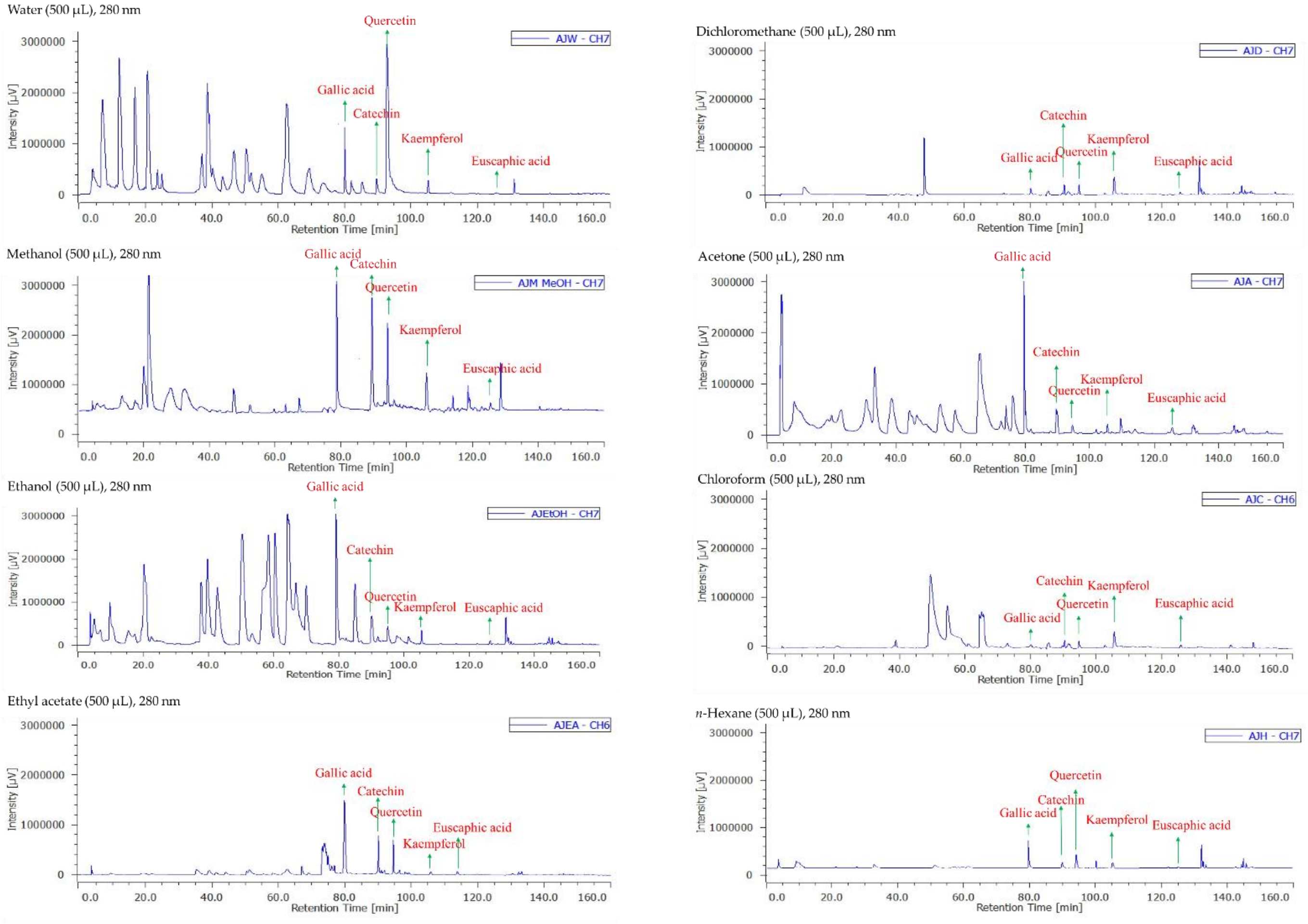

Figure S17. Reverse-phase HPLC chromatogram of all solvent extracts with the same scale.
